# The NIRS Brain AnalyzIR Toolbox

**DOI:** 10.3390/a11050073

**Published:** 2018-05-16

**Authors:** Hendrik Santosa, Xuetong Zhai, Frank Fishburn, Theodore Huppert

**Affiliations:** 1 Department of Radiology, University of Pittsburgh, Pittsburgh, PA 15213-2536, USA; 2 Department of Bioengineering, University of Pittsburgh, Pittsburgh, PA 15213-2536, USA; 3 Department of Psychiatry, University of Pittsburgh, Pittsburgh, PA 15213-2536, USA; 4 Departments of Radiology and Bioengineering, University of Pittsburgh, Clinical Science Translational Institute, and Center for the Neural Basis of Cognition, Pittsburgh, PA 15213-2536, USA

**Keywords:** Functional near-infrared spectroscopy, toolbox, statistical analysis

## Abstract

Functional near-infrared spectroscopy (fNIRS) is a noninvasive neuroimaging technique that uses low-levels of light (650–900 nm) to measure changes in cerebral blood volume and oxygenation. Over the last several decades, this technique has been utilized in a growing number of functional and resting-state brain studies. The lower operation cost, portability, and versatility of this method make it an alternative to methods such as functional magnetic resonance imaging for studies in pediatric and special populations and for studies without the confining limitations of a supine and motionless acquisition setup. However, the analysis of fNIRS data poses several challenges stemming from the unique physics of the technique, the unique statistical properties of data, and the growing diversity of non-traditional experimental designs being utilized in studies due to the flexibility of this technology. For these reasons, specific analysis methods for this technology must be developed. In this paper, we introduce the NIRS Brain AnalyzIR toolbox as an open-source Matlab-based analysis package for fNIRS data management, pre-processing, and first- and second-level (i.e., single subject and group-level) statistical analysis. Here, we describe the basic architectural format of this toolbox, which is based on the object-oriented programming paradigm. We also detail the algorithms for several of the major components of the toolbox including statistical analysis, probe registration, image reconstruction, and region-of-interest based statistics.

## 1. Introduction

Functional near-infrared spectroscopy (fNIRS) is a non-invasive technique that uses low levels of red to near-infrared light (650–900 nm) to measure changes in the optical properties of tissue; particularly those due to changes in blood/hemoglobin volume and oxygenation [1–3]. For brain imaging, fNIRS uses discrete light emitters and detectors (collectively termed optodes) placed on the surface of the scalp either directly or through fiber optics to measure changes in the absorption of light as it passes along a diffuse path between the two optodes. By positioning multiple optodes around the head, different brain regions can be simultaneously recorded and used to infer underlying brain activity based on the brain’s localized hemodynamic response. In comparison to other modalities, such as functional magnetic response imaging (fMRI), fNIRS is more portable, has a lower operating cost, and allows for measurements from a wide range of populations such as those with contraindications to MRI. However, a limitation of this method is its lower spatial resolution and depth of penetration compared to fMRI. FNIRS is limited to the outer cortex of the brain (roughly 5–8 mm of the brain’s surface) [4], but this sensitivity also varies according to subject anatomy. In addition, since these measurements are recorded from the scalp’s surface, fNIRS is also more sensitive to contamination from superficial physiology in the skin, which poses unique challenges for data analysis [5,6].

Over the last three decades, fNIRS has been used in a wide range of studies including pediatric populations (e.g., [7]), clinical studies (e.g., [8]), multimodal validations (e.g., [9]), and cognitive testing (e.g., [10]). However, as the fNIRS field has evolved and this technology has found more acceptance, the complexity of the scientific questions being asked of fNIRS data has dramatically increased. Group-level comparisons, longitudinal analysis, or complex comparisons between different task events are now status quo. In addition, fNIRS studies have been expanded to child and infant populations (reviewed in [7]), to allow a range of motion (including fNIRS studies of brain activity during gait or balance [11]), and more “real-world” experience (reviewed in [12]). However, these studies create challenges to analysis such as the need to deal with complex sources of motion and/or physiological noise artifacts. To date, the majority of fNIRS studies using tools and methods have either borrowed from other fields (primarily functional MRI) or have used modality-agnostic methods such as ordinary least-squares regression or methods coded in general programs such as statistical package for the social sciences (SPSS) [13] or statistical analysis system (SAS) [14]. However, in general these methods are not designed to address the specific and unique features of fNIRS data and thus these make some assumptions that may not be optimal for the non-ideal noise structures and types of artifacts typically present in fNIRS signals.

As recently reviewed by Huppert [15], fNIRS data and its sources of noise have unique properties that require adjustment of the statistical methods in order to accurately control type-I error (false positive). In particular, fNIRS has spatially and temporally structured noise and artifacts [10,15]. Additionally, the typically sparse fNIRS measurement probe geometries have non-uniform measurement profiles and are sensitive to placement, registration, and individual anatomy. These can vary with repeated measurement sessions, longitudinal studies (e.g., child development), and are potentially biased across populations (e.g., systematic differences in brain structure or brain atrophy across two subject groups). Finally, due to factors such as the variability in anatomy of the head and optode coupling through hair, there is often a high-degree of the variability in statistical power between subjects or different spatial channels within the same fNIRS probe. It is not uncommon to observe a several-fold difference in the signal-to-noise ratio in measurements between areas with little hair (e.g., the forehead) and those with hair or thicker bone structure (e.g., the occipital). The use of statistical models whose assumptions do not match these properties often results in unacceptable false-discovery and uncontrolled type-I errors. As our group has reviewed in several recent publications [9,10,15–18], these noise features and unique statistical properties of fNIRS data need to be properly considered and will be briefly summarized in this publication in the context of a new fNIRS analysis toolbox.

The primary rationale for the development of the AnalyzIR (pronounced “an-a-lyze-er”) toolbox was to create a statistical analysis package to specifically address the properties of fNIRS data. This toolbox was designed to capture and preserve as much of this fNIRS-specific information and noise as possible through the entire analysis pipeline such that first- and higher-level statistical analysis methods could use this information in statistical models by utilizing covariance whitening, accounting for dependent noise terms, and using robust statistical methods. For example, the spatial noise due to structured physiological noise between channels of fNIRS data from the first-level (or single subject) statistical models is preserved and accounted for in later region-of-interest or image reconstruction analysis modules. The estimate of first level noise is also used to create whitened and weighted second-level (group) statistical models. Preserving and using these noise structures is important to fNIRS, since as previously mentioned, these can vary considerably across sensor positions or across subjects. Furthermore, one of the key features of this toolbox is a framework for performing sensitivity-specificity (receiver operator characteristics; ROC) analysis to compare analysis methods and pipelines. The current toolbox interfaces to both the popular HOMER-2 [19] and NIRS-SPM [20] fNIRS analysis packages, which allows a head-to-head comparison of various analysis options.

## 2. Architecture of Toolbox

The AnalyzIR toolbox is an open-source analysis package. This toolbox utilizes both custom namespace and class definitions written in MATLAB (MathWorks, Natick MA USA) language to provide an object-oriented programming interface to performing fNIRS analysis. The toolbox is maintained on a public BitBucket.org project (Atlassian Corp. Sydney Australia. www.bitbucket.org/huppertt/nirs-toolbox) as well as the NIH’s NeuroImaging Tools & Resources Collaboratory (NITRC) (https://www.nitrc.org/projects/AnalyzIR). In addition, several demos or examples (e.g., fNIRS analysis, connectivity, group analysis, image reconstruction, registration, etc.) with explanations are provided in the toolbox download.

The AnalyzIR toolbox requires MATLAB version 2014b or higher due to required support of several data types such as the MATLAB table class and graphics modules. However, most core functionality is written to only use the basic MATLAB program without the requirement for additional toolbox libraries. Parts of the toolbox interface to fNIRS forward models (models of the light propagation through tissue/brain) require separate download of the software such as NIRFAST (Near-Infrared light transport in tissue and image reconstruction) [21,22], Matlab/Octave-based mesh generation toolbox (Iso2mesh) [23], MCextreme (Monte Carlo Extreme) [24], MMC (mesh-based Monte Carlo) [25], or the tMCimg software (Monte-Carlo photon transport) [26] packages.

The toolbox currently also includes several functions for generating synthetic or semi-synthetic (experimental baseline data with synthetically added “evoked” responses) data for testing purposes and offers several example scripts and tutorials including several full datasets that can be downloaded with the code.

### 2.1. Data Classes

**Figure 1.**
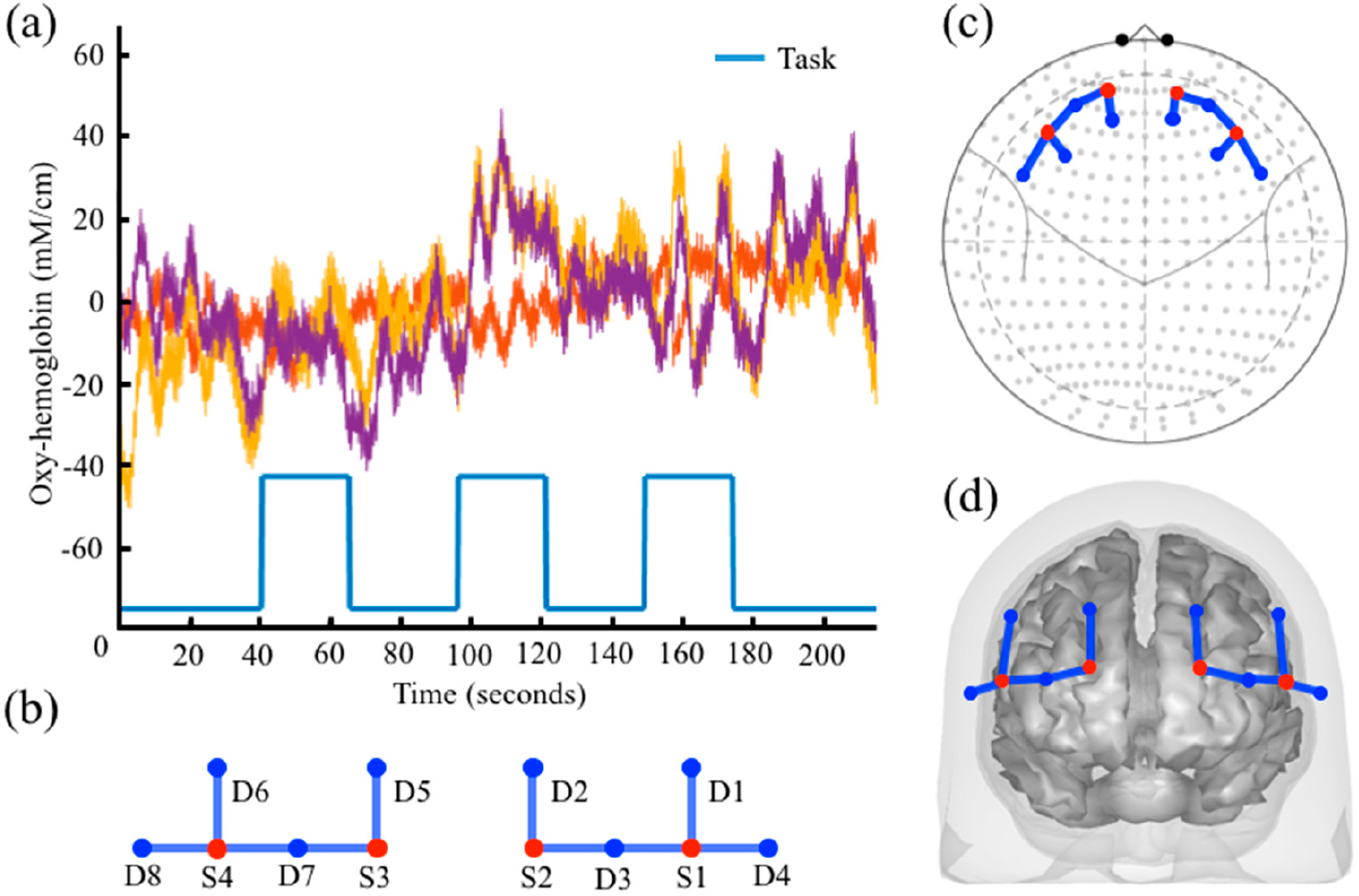
Example plot for data objects: (**a**) Example time series from 3 fNIRS channels with stimulus information shown along the bottom. An example of the same probe object shown in (**b**) 2D probe geometry; (**c**) 10–20 International System, and (**d**) registered 3D probe geometry is also demonstrated.

The framework of the AnalyzIR toolbox is based around an object/class-oriented programming that defines several custom class definitions in the Matlab framework. These objects are self-contained representations of the fNIRS data and contain all the information needed to pass between processing modules. This makes bookkeeping of an fNIRS dataset for analysis easier and provides a very easy framework to add, merge, or remove subsets of a dataset in analysis. These objects can be stacked into arrays or matrices and concatenated or deleted by index using object arrays. Object classes define context-specific methods, such as the drawing commands, which allow the same command (e.g.,) “draw” to act differently depending on the type of object (e.g., time-course, statistics variable, or reconstructed image) that is called upon (see Figure 1). Table 1 provides a partial list of the core data classes and their purpose, methods, and description. This object-oriented coding makes the programming environment very flexible and easy to use as only a few basic terms are needed. There are currently several core data types in the AnalyzIR toolbox, which are detailed below.

#### 2.1.1. nirs.core.Data

The *Data* class defines the object to hold fNIRS time-series information. This object contains a data matrix variable that holds the channel-by-time information of the fNIRS measurements. The *Data* class also holds both stimulus/event timing information and demographics information about that specific data entry. The stimulus information encodes either discrete task events (termed *nirs.design.StimulusEvent* objects in the program) or continuous vector regressors (*nirs.design.StimulusVector* objects), which can encode additional continuous-time regressors in the study. Stimulus events can carry metadata about the onsets, durations, and amplitude modulation of discrete events. This allows specification of various linear models for first-level statistical analysis including linear- and quadratic parametric-modulation of events based on metadata such as reaction time encoded in the event amplitude fields. Stimulus vector models can be used to encode external regressors such as independent measurements of systemic physiology or short-separation fNIRS data as nuisance regressors. The demographics fields on the *Data* class hold categorical or continuous metadata about the subject ID, gender, age, etc. which is populated upon loading of the data (depending on the instrument/company) or imported from a spreadsheet. The inclusion of the demographics information directly on the *Data* class allows bookkeeping to track the data and is later used in second-level analysis modules.

The *Data* class can hold any type of NIRS data including continuous-wave, frequency-domain, and hyper-spectral data. The same class and methods are used to store various time-series data along the full processing pipeline from raw signals to hemoglobin and region-of-interest traces, which allows full flexibility in the use of processing models such as the general linear model which can be applied to any level of processing. *Data* objects can be plotted from command line methods by evoking the “draw” method as shown in Figure 1 or through the time-series viewer (called *nirs.viz.nirsviewer*) included in the toolbox, which provides a “HOMER”-like [19] graphical interface and user interaction.

#### 2.1.2. nirs.core.Data

The *Probe* class is used to store information about the fNIRS head cap layout and registration. This also encodes the source/detector identities of the individual channels of data. The *Probe* class controls its own drawing methods, which allows context-specific behavior for drawing statistical maps on several representations of probes (e.g., flat, 10–20 mapped, 3D registered overlays on the brain or scalp, or region-of-interest bar charts, which is controlled by the object’s *default_draw_fcn* field). The *Probe* class is reused in the *Data*, *Statistics*, and *Image* object classes to provide a common interface for these data types. Probes can be registered to individual or atlas based anatomical MRI within the toolbox or imported from HOMER-2/AtlasViewer [19] and NIRx data formats. Figure 1b–d demonstrates the *probe.draw* function for flat, 10–20, and 3D mesh drawing modes.

#### 2.1.3. nirs.core.ChannelStats

Both first- and higher-level channel-based statistical analysis are encapsulated in the general *ChannelStats* class object. The *ChannelStats* class is created as the result of either the first-level general linear model (GLM) modules or the second (or higher) level group-level models and contains the estimates of “brain activity” for the various task conditions as well as their uncertainty covariance models. Statistical estimates within the class are computed by Student’s *t*-statistic estimates. False-discovery rates are controlled by a Benjamini–Hochberg [27] correction applied to all data contained within the class. Thus, if the class contains multiple fNIRS source-detector pairs, oxy-/deoxy-hemoglobin, and multiple conditions, the correction will be very conservative over all of these estimates. This can be relaxed by separating the task conditions via calls to the “*t*-test” function or by removing the data types to separate oxy- and deoxy-hemoglobin. This class can hold any data type (raw, optical density, hemoglobin, or derived measures such as oxygen saturation or metabolism estimates).

The “*t*-test” function can be evoked from the *ChannelStats* class to create composite statistics and is returned in a similar *ChannelStats* object. The *t*-test is computed by the expression

(1) $$t=c\cdot \beta /\sqrt{c\cdot {Cov}_{\beta }\cdot c^{T}}$$

where c is the contrast vector, β is the activity strength, and T is the transpose operator. For example, if there were five total task conditions, then the test of condition #1 > condition #2 would be given by $c={\left[1-1000\right]}^{T}$. In the toolbox, the contrast can be specified directly as a vector or by human-readable strings; e.g., *MyStats.ttest([1–1000])* or *MyStats.ttest(“task1–task2”)* where “task1/2” are the names of two event types in the data.

The *ChannelStats* object also retains information about the linear model that was used to create it, which allows a hemodynamic response curve to be returned using the function call of form *<MyStats>.HRF* which returns the estimated time course of the hemodynamic response as a *nirs.core.Data* variable. Block averaging, deconvolution, nonlinear, and canonical forms of the first level regression model are all handled.

In addition, the statistics contained within the *ChannelStats* class may also be recalled using the command “table” (e.g., *MyStats.table*), which returns a table of the regression coefficients, *t*-statistics, *p*-values, Benjamini-Hochberg FDR-corrected *p*-values (termed *q*-values), and an estimate of the type-II power for that entry of data. This table can be saved to an Excel or SPSS format or copied to the virtual clipboard. The type-II power calculation is estimated by computing the minimum detectable change (MDC) [28] and given by the expression

(2) $$MDC=\left(T_{\alpha }(n+m-2)+T_{2\beta }(n+m-2)\right)\cdot \sqrt{\frac{MSE}{n}+\frac{MSE}{m}}$$

where $T_{\alpha }(n+m-2)$ and $T_{2\beta }(n+m-2)$ are the Student’s *t*-value for the type-I error (α) and statistical power (1-β) control with n+m-2 degrees-of-freedom. The MDC defines the value of the change in the fNIRS signal needed to reach a specific power to reject the null hypothesis at a given alpha. Given an experimentally measured value, we can use the inverse of this equation to solve for the power (1-β). For example, given a measured Student’s *t*-value of T(998,1)=3.0 for the fNIRS signal change $\left(=MDC/\sqrt{\frac{MSE}{n}+\frac{MSE}{m}}\right)$ with n=m=500, then $T_{\alpha }\;(998,1)=1.64$ at α=0.05. Solving for the power, (1-β)=0.82 or the measurement is 82% powered to detect a p<0.05 change for the one-sided test. For both first- and higher-level models, the power for an fNIRS measurement is returned within the *ChannelStats* object using this equation. Compared to other modalities, such as fMRI, where the measurement error (and therefore power) is more uniform across space or participants, the power in fNIRS measurements can vary significantly across the probe or between subjects with more or less hair under the fNIRS sensors.

In addition, the *ChannelStats* variable can also compute multi-variate contrast (e.g., oxy- and deoxy-hemoglobin joint estimates) using a Hotelling’s $t^{2}$ test [29]. $t^{2}$ statistic is defined as

(3) Tp,n-12=(n-p-1)npβTΣ^-1β

where β is the vector of regression coefficients and Σ is the covariance of these estimates both spanning across data-types (e.g., oxy-/deoxy-hemoglobin covariance). Thus, this $T^{2}$ value is used within the toolbox to define contrast across hemoglobin species (e.g., the null hypothesis of no change in signal given the joint probability of oxy- and deoxy-hemoglobin) and follows a F(p,n-1) statistical distribution. This value is stored in a related object class called *ChannelFStats* is also contained within the toolbox, which is also used in the higher-level ANOVA models.

#### 2.1.4. nirs.core.ImageStats

Similar to the *ChannelStats* object class, *ImageStats* holds statistical parametric maps following image reconstruction and is created by one of the reconstruction modules. *ImageStats* objects contain the statistics associated with the reconstructed single subject or group-level data into brain space and are displayed by specification of both the type-I error control (e.g., p<0.05) and type-II power (e.g., β>0.80). The later is based on the estimate of the minimum detectable change Equation (2) using the optical forward model (measurement sensitivity) and can be used to delineate the edges or blind spots of the fNIRS probe. Section 4 of this work is devoted to further details of the forward and inverse models and *ImageStats* variable class.

#### 2.1.5. Multimodal Object Classes

While the AnalyzIR toolbox is written primarily for fNIRS data, it does provide limited support for electroencephalography (EEG), magnetoencephalography (MEG), and surface-based fMRI (connectivity informatics technology initiative; CIFTI) dense time-series data [30]). Most of the NIRS data object classes are either reused or slightly modified for this multimodal data (e.g., *nirs.core.ChannelStats* → *eeg.core.ChannelStats*), which allows for most of the processing modules and utility functions to operate on any type of data. Most importantly, the similarity of the object definitions allows the development of multimodal methods, such as joint Bayesian image reconstruction [31–33] and other multimodal statistical and fusion methods.

### 2.2. Processing Modules Classes

Within the AnalyzIR toolbox, data analysis is performed by a collection of processing modules, which are encapsulated within an abstract *nirs.modules* class definition. Processing modules can be concatenated together to create analysis pipelines, which automatically feed the output of upstream modules forward. Table 2 provides a list of the basic processing modules and their citations as used in the toolbox. It is noted that there are more functions/modules in the AnalyzIR toolbox, which are not including in Table 2. Processing modules are chained together to form analysis pipelines, which route the output of one module into the next.

#### 2.2.1. Data Management

Several processing modules are designed for data management including renaming, removing, or splitting stimulus conditions, importing demographics information to data variables, and editing stimulus timing information.

#### 2.2.2. Pre-Processing

The toolbox offers several standard basic pre-processing modules including resampling, conversion to optical density and the modified Beer-Lambert law. In addition, there are several more unique processing modules implemented which are detailed below as a reference to the method.

##### 2.2.2.1. Baseline Correction

The baseline correction algorithm is designed to remove statistical outliers in the innovations of the data following an autoregressive integrative (ARI) model fit of the data. In this code, a *p*th-order ARI (p,1) model is first fit to each channel of fNIRS data,

(4) $$\left(Y\left(k\right)-Y\left(k-1\right)\right)=\sum_{i=1}^{p}a_{i}\cdot \left(Y\left(k-i\right)-Y\left(k-1-i\right)\right)+innov\left(k\right)$$

where k is the index of the measurement sample. The optimal model order (p) is computed from a Bayesian information criterion with a default maximum of 8 times the sample rate (e.g., 8 × 10 Hz = 80 samples). The integrative order is set at 1 and is not adjusted. Based on the estimated model coefficients, the innovations time course is extracted. The innovations of the model are the unique information “injected” at each time point, which is not predicted by the autoregressive history of the data. As described in our work on AR pre-whitening of the GLM model [16] both spike and shift variations of motion artifacts in fNIRS data are often statistical outliers in this innovations space. Although not all motion artifacts will be outliers, fixing non-statistical outliers are less relevant for the model. From the studentized innovations, a weighting function is computed using the Huber bisquare function

(5) $$w\left(\frac{inn}{\sigma }\right)=\left\{\begin{array}{l} 1-{\left(\frac{inn}{\sigma \cdot \kappa }\right)}^{2}\\ 0 \end{array}\left|\begin{array}{c} \frac{inn}{\sigma }\\ \frac{inn}{\sigma } \end{array}\right|\begin{array}{c} <\kappa \\ \geq \kappa \end{array}\right.$$

where the weight (w) varies from 0 (discard; definitely an outlier) to 1 (keep), σ is the standard deviations of the errors, and κ is the tuning constant (κ=4.685 for the bisquare produce 95% efficiency when the errors are normal). This weighting is then applied to the innovations and the autoregressive process is then applied in the forward direction to recover the filtered original time-course.

##### 2.2.2.2. PCAfilter

The PCAfilter module within the toolbox implements a spatial principal component filter. There are flags on the module to control the behavior of this filter to correct for motion (components computed across all data-types) or physiology (components computed within data-types from either the entire same file, the baseline periods of the same file, or a separate baseline file). The PCA filter is implemented as described in Zhang et al. 2005 [38]. The removed components can either be defined directly based on the number of components or based on a fraction (e.g., 80%) of the spatial covariance to remove. A related module called Kurtosis filter, uses the same PCA approach but defines the components to down-weight or remove based on the estimated kurtosis of the component time-course. A Huber bisquare function Equation (5) is used to down-weight components with high Kurtosis.

#### 2.2.3. Calculate CMRO_2_

In addition to raw signal intensity and oxy-/deoxy-hemoglobin time traces, the *nirs.core.data* data class is able to store any arbitrary derived time-course data such as total-hemoglobin, tissue oxygen saturation (S_t_O_2_), relative pulsatile blood flow (pCBF) [39], and estimates of the cerebral metabolic rate of oxygen (CMRO_2_). The CMRO_2_ module within the toolbox pipeline supports both a steady-state and dynamic model of metabolism. The CMRO_2_ models are given by the two models:

Steady state Equations [40]:

(6a) $$q(t)=1+\frac{\Delta Hb(t)}{OEF_{0}\cdot HbT_{0}}$$

(6b) $$v(t)=1+\frac{\Delta Hb(t)\;+\;\Delta HbO_{2}(t)}{HbT_{0}}$$

(6c) $$f(t)=v{\left(t\right)}^{\frac{1}{\alpha }}$$

(6d) $$OEF(t)=\frac{q(t)}{v(t)}+\frac{\tau }{f(t)}\cdot \left(\frac{dq(t)}{dt}-\frac{q(t)}{v(t)}\cdot \frac{dv(t)}{dt}\right)$$

(6e) $${CMRO}_{2}(t)=f(t)\cdot OEF(t)$$

Dynamic Equations [41]:

(7a) $$\frac{dI_{flow}(t)}{dt}=a_{0}\cdot U_{flow}(t)-\tau_{s}\cdot I_{flow}(t)-\tau_{feedback}\cdot (f(t)-1)$$

(7b) $$\frac{dI_{{CMRO}_{2}}(t)}{dt}=b_{0}\cdot U_{{CMRO}_{2}}(t)-\tau_{s}\cdot I_{{CMRO}_{2}}(t)-\tau_{feedback}\cdot \left({CMRO}_{2}(t)-1\right)$$

(7c) $$\frac{df(t)}{dt}=I_{flow}(t)$$

(7d) $$\frac{d{CMRO}_{2}(t)}{dt}=I_{{CMRO}_{2}}(t)$$

(7e) $$\frac{dv(t)}{dt}=\frac{1}{\tau }\cdot \left(f(t)-v{\left(t\right)}^{\frac{1}{\alpha }}\right)$$

(7f) $$\frac{dOEF(t)}{dt}=\frac{\left(f(t)\cdot I_{{CMRO}_{2}}(t)\;-\;{CMRO}_{2}(t)\cdot I_{flow}(t)\right)}{f{\left(t\right)}^{2}}$$

(7g) $$\begin{array}{rr} & \frac{dq(t)}{dt}=\frac{1}{\tau }f(t)\cdot \left(OEF(t)-\frac{q(t)}{v(t)}\right)+\frac{q(t)}{v(t)}\cdot \frac{dv(t)}{dt} \end{array}$$

In both of these models, q(t), v(t), and f(t) are the normalized deoxy-hemoglobin $\left(Hb(t)/Hb_{0}\right)$ and blood volume/total hemoglobin $\left(HbT(t)/HbT_{0}\right)$, and blood flow signals respectively. OEF(t) is the oxygen extraction fraction $\left(OEF=\left(S_{a}O_{2}-S_{v}O_{2}\right)/S_{a}O_{2}\right.$ where $S_{a}O_{2}$ and $S_{v}O_{2}$ are the arterial and venous oxygen saturations). Further details of these models can be found in Hoge et al. [40] and Rierra et al. [41]. These two model versions are then fit to the fNIRS data using the option of either a nonlinear search method or an extended Kalman filter implementation. The output of these modules is an estimate of the state variables (flow, CMRO_2_) as time-course variables in the toolbox. Using these models, oxygen metabolism, oxygen extraction fraction, and blood flow can be estimated as hidden state variables from fNIRS measurements of the changes in oxy- and deoxy-hemoglobin under the assumptions of the two models. It is important to note that these are not directly measured variables and users are strongly encouraged to background in the Hoge et al. [40] and Rierra et al. [41] in order to understand the limitations of these models. Although some research shows that these simplified models may not estimate oxygen metabolism as accurately when compared to more detailed multiple compartment models [42–44], these models may provide some basis to interpret the relationships of oxy- and deoxy-hemoglobin signals.

#### 2.2.4. HOMER-2 Interface

The AnalyzIR toolbox contains a module for evoking many of the functions of the external HOMER-2 NIRS toolbox [19]. This provides access to many of the pre-processing tools that are not included directly in our toolbox. This module contains the means to translate many of the keywords used in the HOMER-2 code (such as sample rate or data-type access) such that most of the bookkeeping to use the HOMER-2 code is accounted for through our toolbox. This also uses internal protected variables to pass options between HOMER-2 functions, such that entire pipelines can be built around the HOMER-2 functionality. Due to major differences in the handling of statistical variables, only the pre-processing and filtering HOMER-2 modules are supported. A generic MATLAB wrapping module is also included to call any arbitrary function as part of an AnalyzIR toolbox pipeline.

## 3. Statistical Modules

The AnalyzIR toolbox contains a number of processing modules for statistical analysis for both first level (single scans) and higher-level models. These include time-series regression models, functional connectivity models, image reconstruction methods, and higher order mixed effects and ANOVA models.

### 3.1. First-Level Statistical Models

In many fNIRS experimental studies, the fNIRS signals between each source-to-detector pair are analyzed using a general linear regression model to test for statistical differences between the baseline and task conditions for each scan. This approach is similar to functional MRI, although several differences in the structure of noise in fNIRS compared to other modalities should be noted (see [15] for review).

In general, first level statistical models for examining evoked signal changes are given by a regression model described by the equation

(8) Y=X×β+ϵ

where X is the design matrix encoding the timing of stimulus events, β is the coefficient (weight) of that stimulus condition for that source-detector channel, and Y is the vector of measurements. The design matrix (X) can come from either a canonical model of the expected response or a deconvolution model (see Section 3.2).

#### 3.1.1. OLS

The ordinary least-squares (OLS) processing module will solve Equation (8). This is the model historically implemented in the HOMER and HOMER-2 toolboxes [19]. The OLS solution to Equation (8) is given by

(9a) $$\beta ={\left(X^{T}\cdot X\right)}^{-1}X^{T}\cdot Y$$

(9b) $${Cov}_{\beta }={\left(X^{T}\cdot X\right)}^{-1}\cdot \sigma^{2}$$

(9c) $$\sigma^{2}={\left(Y-X\times \beta \right)}^{T}\cdot (Y-X\cdot \beta )$$

#### 3.1.2. AR-IRLS

As detailed in Huppert 2016 [15], two of the issues in fNIRS noise are serially-correlated errors and heavy-tailed noise distributions due to slow systemic physiology and motion-related artifacts respectively. Our previous work in Barker et al. 2013 [16] described an autoregressive pre-whitening approach using iteratively reweighted least-squares (AR-IRLS) to control type-I errors in the fNIRS statistical model. In brief, this regression model uses an *n*-th order auto-regressive filter $\left(W_{AR}\right)$ determined by an Akaike model-order (AIC) selection to whiten both sides of this expression, e.g.,

(10) $$W_{AR}\times Y=W_{AR}\times X\times \beta +W_{AR}\times \varepsilon$$

As described in Barker et al. 2013 [16], the regression model is first solved using robust regression and the residual noise is then fit to an AR model. This filter $\left(W_{AR}\right)$ is applied to both sides of the original model and then resolved and repeated until convergence. This AR filter alleviates serially correlated errors in the data that result from physiological noise and/or motion artifacts. AR whitening, however, does not address the heavy-tailed noise from motion artifacts. To do this, the AR-whitened model is solved using robust weighted regression, which is a procedure to iteratively down-weight outliers such as motion artifacts.

(11a) $$S\cdot W_{AR}\times Y=S\cdot W_{AR}\times X\times \beta +S\cdot W_{AR}\times \varepsilon$$

where S is

(11b) $$S\left(\frac{r_{W}}{\sigma }\right)=\left\{\begin{array}{ll} 1-{\left(\frac{r_{W}}{\sigma \cdot \kappa }\right)}^{2} & \left|\frac{r_{W}}{\sigma }\right|<\kappa \\ 0 & \left|\frac{r_{w}}{\sigma }\right|\geq \kappa \end{array}\right.$$

which is simply the square root of Tukey’s bisquare function [45] and is the same model as used in Equation (4) from Barker et al. [16]. The tuning constant κ is typically set to 4.685 which provides 95% efficiency of the model in the presence of normally distributed errors σ is the standard deviation of the residual noise in the model.

Using this model, the regression coefficients (β) and their error-covariance is estimated, which is used to define statistical tests between task conditions or baseline. The regression model is solved sequentially for each data file for each subject. All source-detector pairs within a file are solved concurrently yielding a full covariance model of the noise, which is used in group-level and region-of-interest analysis to correct for the spatial inter-dependence of measurement channels. The estimate of β and its covariance matrix is given by the expressions

(12a) $$\beta ={\left(X^{T}\cdot W_{AR}^{T}\cdot S^{T}\cdot S\cdot W_{AR}\cdot X\right)}^{-1}\cdot X^{T}\cdot W_{AR}^{T}\cdot S^{T}\cdot S\cdot W_{AR}\cdot Y$$

(12b) $${Cov}_{\beta }={\left({\left(W_{AR}\cdot X\right)}^{T}\times W_{AR}\cdot X\right)}^{-1}\cdot \sigma^{2}$$

(12c) $$\sigma^{2}={\left(W_{AR}\cdot Y-W_{AR}\cdot X\times \beta \right)}^{T}\cdot \left(W_{AR}\cdot Y-W_{AR}\cdot X\cdot \beta \right)$$

#### 3.1.3. NIRS-SPM

The GLM module can access the model implemented as part of NIRS-SPM [20]. The NIRS-SPM toolbox must be separately downloaded from our methods. Details on the NIRS-SPM model are found in [20]. Our toolbox allows interfacing to both the pre-whitening and pre-coloring SPM modules, as well as the autoregressive models and minimum descriptive length (MDL) wavelet [46] approaches. The output of the NIRS-SPM code is modified to be consistent with the *ChannelStats* object class.

#### 3.1.4. Nonlinear GLM

A nonlinear GLM is also implemented to estimate the first level statistical model. In this module, a canonical hemodynamic response function (HRF) model is nonlinearly estimated by iteratively estimating the FIR based regression model using derivative terms for time and dispersion of the canonical model followed by update of the canonical model parameters. The nonlinear basis set is controlled by the same basis set dictionary as the other GLM versions (see Section 3.2), which allows it to be adjusted to use the same shape fit from all data within a single file, separate shapes for different data types (e.g., oxy-/deoxy-hemoglobin), or even allow different shapes and timing for each separate task condition.

### 3.2. Canonical and Basis Sets

There are several basis sets offered within the toolbox as described below. In the first-level statistical model estimators (GLM, OLS, NIRS-SPM modules), the basis set is prescribed to one or more task conditions using a dictionary class. The assignment to “default” will use the basis set for all conditions that are not explicitly assigned a different basis. Oxy- and deoxy-hemoglobin can be assigned different basis sets using an assignment such as “default:hbo”. The default in the toolbox is to use the same basis set (canonical HRF) for all task conditions and data types.

#### 3.2.1. Canonical HRF

The canonical HRF (or double gamma function) is the default basis set in the toolbox and models a hemodynamic response with an undershoot period. This is defined by the equation:

(13) $$HRF=\frac{b_{1}^{a_{1}}\;\times \;t^{\left(a_{1}-1\right)}}{\Gamma \left(a_{1}\right)}\times e^{\left(-b_{1}*t\right)}-c\times \frac{b_{2}^{a_{2}}\;\times \;t^{\left(a_{2}-1\right)}}{\Gamma \left(a_{2}\right)}\times e^{\left(-b_{2}\times t\right)}$$

where $b_{1}$ (default: 1 s^−1^) and $b_{2}$ (default 1 s^−1^) are the dispersion times constants for the peak and undershoot period, and $a_{1}$ (default 4 s) and $a_{2}$ (default 16 s) are the peak time and undershoot time. c (default 1/6) is the ratio of the height of the main peak to the undershoot. Γ(.) is the scalar value of the gamma function and is a normalizing factor. The canonical basis set has the option to include the first derivatives in the regression model, which are computed as a finite difference with respect to the $a_{1}$ and $b_{1}$ variables.

#### 3.2.2. Gamma Function

The gamma HRF basis set models a hemodynamic response without an undershoot period. This is defined by the equation:

(14) $$HRF=\frac{b_{1}^{a_{1}}\;\times \;t^{\left(a_{1}-1\right)}}{\Gamma \left(a_{1}\right)}\times e^{\left(-b_{1}\times t\right)}$$

where $b_{1}$ (default: 1 s^−1^) is the dispersion times constants and $a_{1}$ (default 6 s) is the peak time.

#### 3.2.3. Boxcar Function

The boxcar model uses a constant amplitude block for the duration of the task event. The boxcar model contains two user-defined variables for the lag-time, which shifts the onset of the boxcar (default 3-s) and the IRF-duration (default 5 s), which extends the duration of the event. The regressor is the convolution of the task duration per event with the IRF-duration.

#### 3.2.4. FIR-Deconvolution

The finite impulse response (FIR) model allows an unconstrained deconvolution and estimation of the full hemodynamic response. In this basis set, the “bin width” and “n[umber] bins” must be specified. The bin width defines the temporal resolution of the estimator and is either specified as the number of samples (when *binwidth* is a numeric) or in seconds (when *binwidth* is a string ending in “s” such as “1.5 s”). The *binwidth* allows down sampling of the estimated hemodynamic response by binning sample points in the design matrix (X). The variable *nbins* specifies the number of bins used in the design model such that the total length of the deconvolution window is the nbins * binwidth. This product must be wide enough to fit the entire expected response curve (generally 12–18 s longer then the task duration). A limitation of FIR-deconvolution is that the same window is used for all trials and thus the blocks must have the same task duration. The basis dictionary in the GLM processing module can be used to assign a different FIR basis set to each condition, which allows different windows to be used for different event tasks. The deconvolution results in a large number of “betas” (regression coefficients) to be estimated and stored in the *ChannelStats* variable. The statistical contrast over a window of the response can be estimated using a callback of the form *MyStats.ttest(“taskA[4:10]”)* [for the 4th–10th coefficient] or *MyStats.ttest(“taskA[2:8s]”)* [for the window of 2 s – 8 s] where *MyStats* is the name of the *ChannelStats* variable and taskA is the condition name. When a window of time used in the contrast is a non-integer of the FIR *binwidth*, the contrast vector used is a linear interpretation (e.g., c=[00.20.8111] in Equation (1) (Section 2.1.3)) starts the contrast at 1.8 s or 80% between the 2nd and 3rd sample for the example at a 1 Hz sample rate. The callback *MyStats.HRF* will return a *nirs.core.data* (time-series) variable from the estimated hemodynamic response which can then be drawn to show the response shape.

#### 3.2.5. FIR-Impulse Response Deconvolution

The FIR basis set also can be used to estimate an impulse-response model using the flag “*useIRF*” in the basis set object. In this case, the task duration is used to convolve the deconvolution model by a boxcar of the duration of the event. This reduces the number of coefficients in the model and allows modeling of scans with varied task durations in the same file (e.g., self-paced tasks). In this option, the *nbins* and *binwidth* variables define the window and resolution of the impulse response, which is generally about 12–18 s in duration regardless of the duration of the task event. This model assumes that the hemodynamic response is linearly additive (e.g., a 10 s duration response is the same as two consecutive 5 s durations added together).

#### 3.2.6. General Canonical

The general canonical basis set allows the user to define a custom canonical model by specifying the impulse response (IRF) vector and a time vector corresponding to the sample points of the IRF.

#### 3.2.7. Vestibular Canonical

Previous research by our group using fNIRS to study human vestibular function [47–49] has noted that the hemodynamic response to vestibular stimulation is very elongated compared to the canonical response. A specific basis for modeling these responses is included, which elongates the response by a user-defined parameter (default 40 s).

### 3.3. Parametric Models

**Figure 2.**
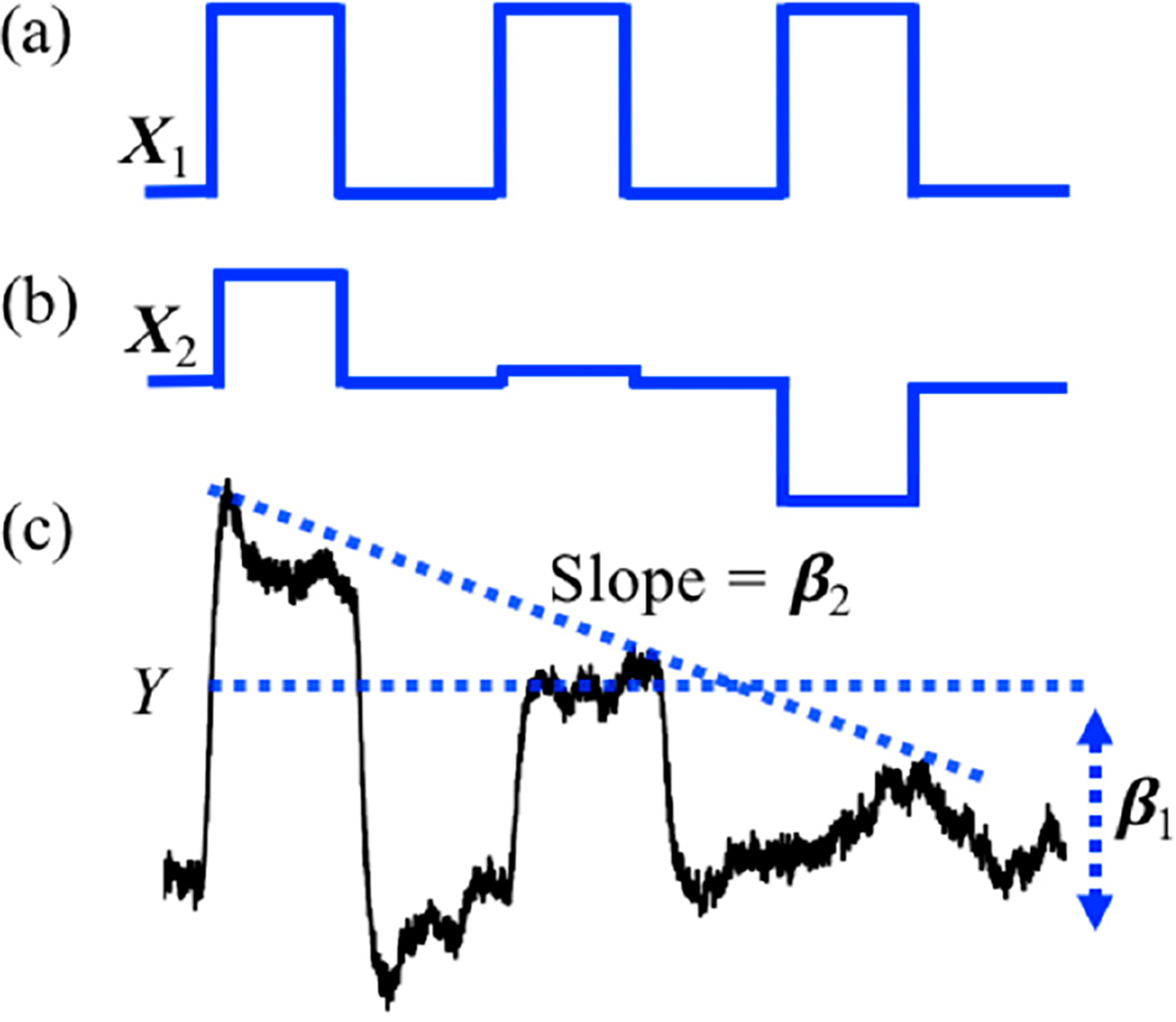
Illustration of the parametric stimulus design: (**a**) Conventional box-car function as the stimulus design; (**b**) parametric stimulus design with time varying models; (**c**) evoked signal from parametric stimulus design in (**b**).

The *StimulusEvents* data class, which holds the onsets and durations of task events, is also used to encode amplitude information that can be used in parametric regression models. In the parametric model, the amplitude of each task block is varied. This can be used to test for brain regions where the amplitude of the brain response covaries with, e.g., reaction time or some other block-dependent variable encoded in the amplitude of the stimulus events. The tool function *nirs.design.split_parametric* is used to construct and manipulate these models. This function requires a formula to be specified, for example “<condition> * (1 + amp + amp^2)” would encode three regressors for (i) a term that is constant for all blocks; (ii) a term that linearly varies by the amplitude parameter across blocks, and (iii) a quadratically varied term. This can be used to specify a condition-specific model where “<condition>” is replaced by the name of the condition in the files. The notation “?” is used to apply the formula to all conditions. The formula supports higher order (including negative) power models as well. The formula can also use the keywords “dur” [duration] and “time” in addition to “amp” [amplitude] to allow models where the regressors vary over the experiment time (within each scan) or by task duration. The function also takes a flag for centering the variables (amplitude, time, or duration) in the model, which subtracts the mean of the variable before encoding the model such that the DC-term (e.g., <condition>*1) part of the model represents the average response. An example of the design model returned by the *split_parametric* function is shown in Figure 2. In this example, a subject repeats a blocked task three times and we wish to test the hypothesis of “did learning occur?” over the blocks. To do this, we use a parametric model modulated by time (e.g., “<condition> * (1 + time)”) which creates two regressors in the GLM model. The first regressor shown in Figure 2a represent the conventional stimulus information produced by a box-car function and models brain activity that stays the same for all three trials. The second regressor shown in Figure 2b is modulated by time and models brain activity is higher for the first block then the last block. Figure 2c shows the example of hemodynamic response generated by convolving the parametric stimuli pattern in Figure 2b and the simulated raw signal. When this model is solved, the second regressor can be used to test the null hypothesis of whether the event blocks did not change linearly over time (e.g., was there a learning effect?). The toolbox has the option to mean center the variables (default is true as used in this example).

### 3.4. Comparison of Models

**Figure 3.**
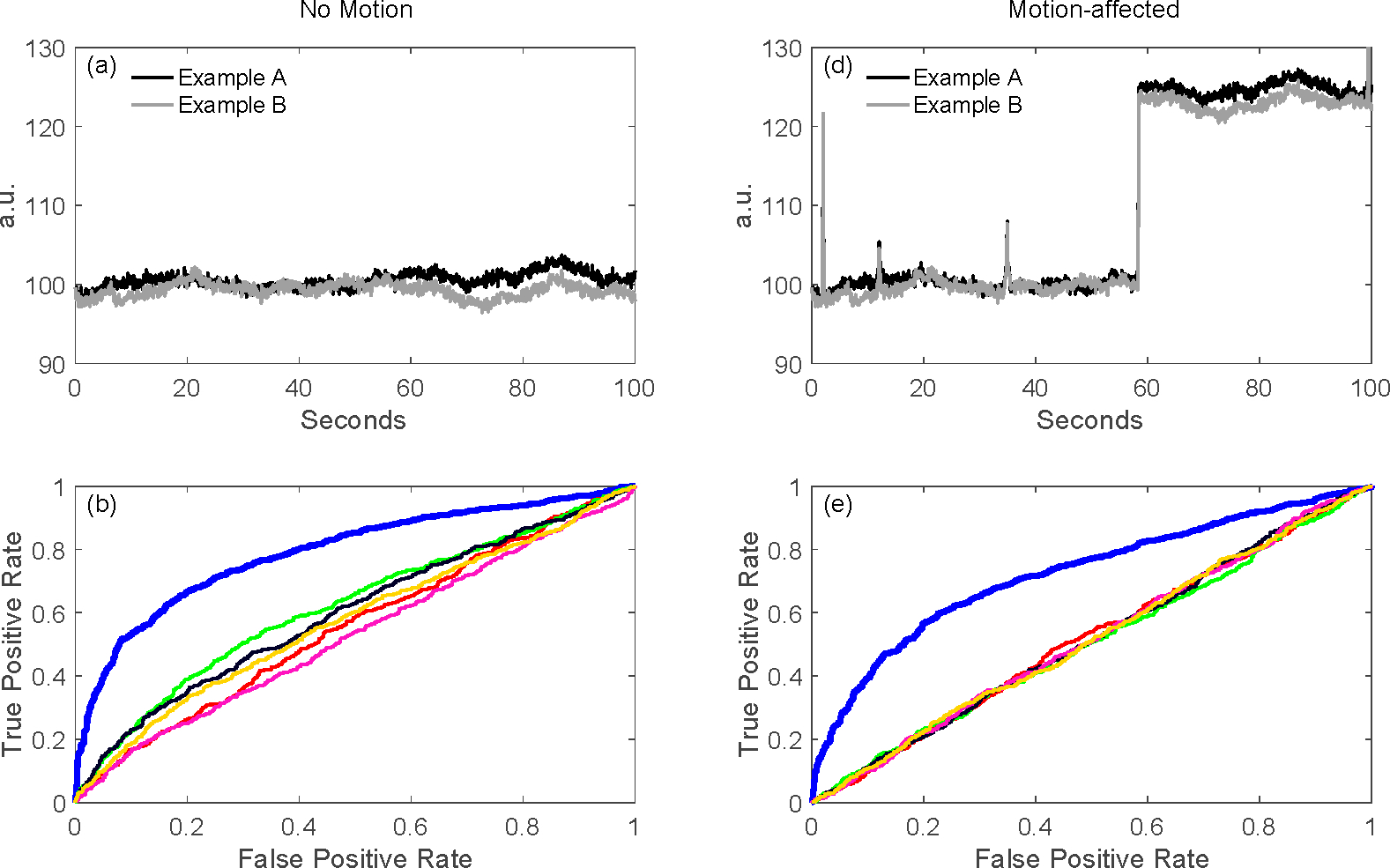

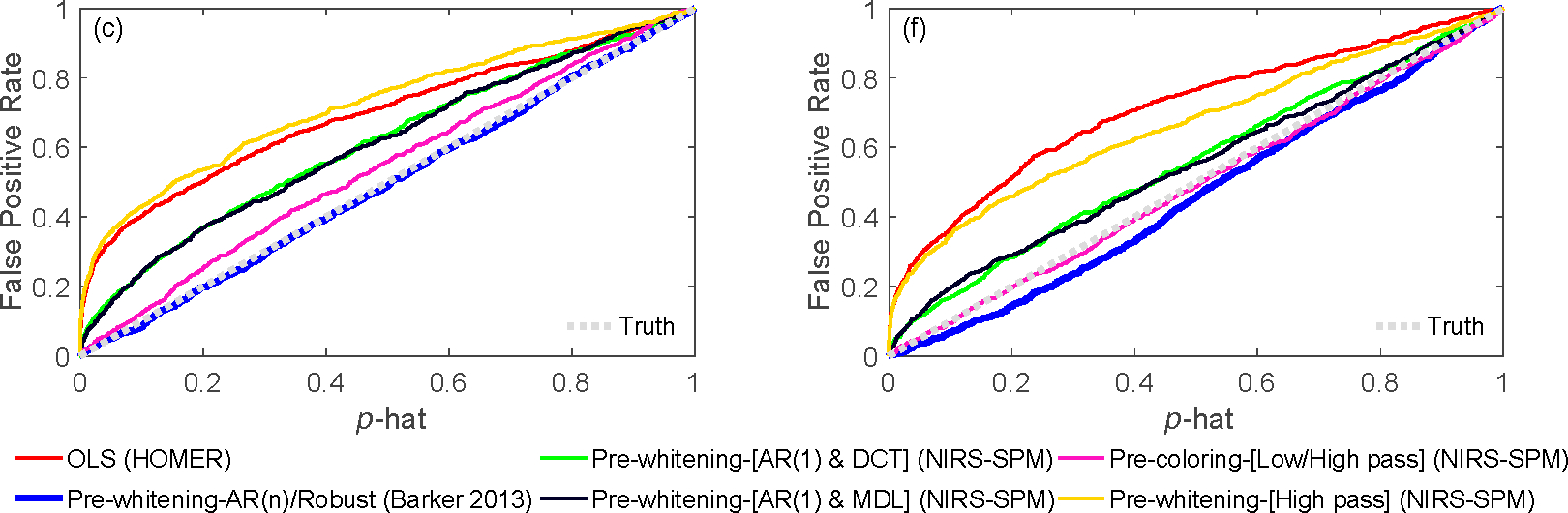
General linear model (GLM) comparison of no motion (left column) and motion-affected (right column). In panels (**a**,**d**), raw signals with no motion artifacts and with artifacts are shown. After simulations, the ROC curves for the various processing applied to the simulated data are shown in (**b**,**e**). Panels (**c**,**f**) show control for type-I errors for the same data and processing. An ideal curve would be along the diagonal (slope = 1), where the reported and actual FPRs would be the same.

As described later in this paper (Section 6.4), a key feature of our toolbox is the ability to quantitatively compare analysis pipelines using receiver operator curve analysis (ROC). This allows varied configurations of the pipeline to be compared and their performance quantified. We demonstrate this feature in Figure 3 by comparing the performance of the variations of the GLM model described in the previous sections. Using the toolbox’s code for simulating test data (see Section 6.4.1) fNIRS time courses were simulated with physiological auto-regressive noise without (Figure 3a) or with (Figure 3d) motion artifacts. A ROC curve analysis (see Section 6.4) was then run from 200 iterations of each model. In Figure 3b,c,e,f, we show the output of one of the demonstration scripts included in our toolbox comparing the performance of ordinary least-squares (OLS) [19], NIRS-SPM with pre-coloring [20], NIRS-SPM with pre-whitening [20], and our AR-IRLS [16] version of the first-level statistical estimators. Figure 3b,e describe the ROC curves (sensitivity-specificity) with no motion and with motion artifacts, respectively. The two plots types presented show the ROC curve (Figure 3b/e) and the control for type-I error (Figure 3c/f). The control for type-I error plots show the actual false-positive rate (as determined by the ROC curve) plotted against the estimated theoretical error (*p*-hat). The ideal behavior is a line of slope unity meaning the type-I error is properly reported by the model. In the case of OLS and some of the options of the NIRS-SPM models, these lines are above unity indicating that the statistical estimates are over-reporting the significance compared to the ROC curve (e.g., the model reported p=0.05 but the false-positive rate was actually closer to 30%). This has been reviewed in our recent publication [15]. In general, our proposed method of AR-IRLS [16] has higher area under the curve (AUC) than other methods. Figure 3e has lower AUC than Figure 3b due to motion artifacts affected. However, the response of AR-IRLS has the AUC above the chance level (other methods has the AUC about 0.5) as shown in Figure 3e. Figure 3c,f depict the control of type-I error (false positive rate) against the *p*-hat at various threshold from the ROC curves in the second row (Figure 3b,e). For the control of type-I error, AR-IRLS has better control for both no motion and motion-affected than other methods, which is close to the ideal condition (“truth”).

### 3.5. Second-Level Statistical Models

Second-level (e.g., group) models using fixed-effects, mixed-effects, or ANOVA are offered as modules in the toolbox. For all three of these processing modules, a formula is specified to define the statistical model used. This formula is defined in Wilkinson-Rogers notation [50], which is also used in Matlab (e.g., *fitlme.m* function) and R software packages. The statistical model formula is able to use any demographics variables stored in the first-level *ChannelStats* variables such as subject ID, group membership, age, etc. Both categorical and continuous variables are supported in these models. Examples of the Wilkinson–Roger’s notation is shown in Table 3.

As an example, let’s consider a group level model consisting of 20 subjects with a range of ages. Each subject preformed three tasks (low, medium, and high) and therefore there are a total of 60 variables (β; 20 subjects × tasks) from the first-level models. For the moment, we are only dealing with a single source-detector pair to simplify the example. We wish to use a mixed effects model to examine the group response and the effect of age on the brain response. The formula we will use has the form “beta ~−1 + cond + cond × age + (1|subject)”, which encodes a model with a term for each task conditions (independent of the subject age) and a second term capturing how the brain response to the condition covaries according to subject age. We will center the variables in the model (the default option in the toolbox), which means that the age-independent term will represent the brain activity for a subject of the average age in our group. In this formula, the demographics variable “subject” is denoted as a random effect, which will pool across the three conditions for each of the 20 subjects. The group-level mixed effects model is described by the equation

(15) β=A⋅Γ+B⋅Θ+ε

where β is the vector of weights obtained from the first level statistical model entries for each subject, task condition, and source-detector pair. A is the fixed effects model and B is the random effects model matrices. In this example of using inclusion of the age as a cofactor is given by

(15) $$\left[\begin{array}{c} \beta_{Subj_{A}},Cond_{Low}\\ \beta_{Subj_{A}},Cond_{Med}\\ \beta_{Subj_{A}},Cond_{High}\\ \vdots \\ \beta_{Subj_{N}},Cond_{Low}\\ \beta_{Subj_{N}},Cond_{Med}\\ \beta_{Subj_{N}},Cond_{High} \end{array}\right]=\left[\begin{array}{cccccc} 1 & & & age_{A} & & \\ & 1 & & & age_{A} & \\ & & 1 & & & age_{A}\\ & \ddots & & & \ddots & \\ 1 & & & age_{N} & & \\ & 1 & & & age_{N} & \\ & & 1 & & & age_{N} \end{array}\right]\left[\begin{array}{c} {\text{Γ}}_{Low}\\ {\text{Γ}}_{Med}\\ {\text{Γ}}_{High}\\ {\text{Γ}}_{Low\text{:}Age}\\ {\text{Γ}}_{Med\text{:}Age}\\ {\text{Γ}}_{High\text{:}Age} \end{array}\right]+\left[\begin{array}{ccc} 1 & & \\ 1 & & \\ 1 & & \\ & \vdots & \\ & & 1\\ & & 1\\ & & 1 \end{array}\right]\times \left[\begin{array}{c} {\text{Θ}}_{A}\\ \vdots \\ {\text{Θ}}_{N} \end{array}\right]+v$$

where the terms $\Gamma_{low}$, $\Gamma_{med}$, and $\Gamma_{highT}$ denote the main group level effects for the three task conditions and the terms $\Gamma_{X:Age}$ denote the interaction terms between the three conditions and age. The second matrix (B) and coefficients (Θ) denote the random effects terms (here indicating subject as a random effect).

Since the covariance of this model is known from the first level model (Section 3.1), the mixed effects model is then solved for using weighted least-squares regression where a weighting matrix (Ω) is applied to the left and right hand sides of the expression and given by

(17a) Ω⋅β=Ω⋅A×Γ+Ω⋅B⋅Θ+Ω⋅v

where the whitening matrix is defined as

(17b) $$\Omega^{T}\times \Omega ={Cov}_{\beta }^{-1}$$

and ${Cov}_{\beta }$ is the noise covariance matrix given in Equations (9b) and (12b), which was estimated from the temporal general linear model (Section 3.1) Ω is estimated from a singular value decomposition of the symmetric covariance matrix. Note that we have written this expression only for one fNIRS measurement channel for simplicity, but in reality this model also includes all source-to-detector channels simultaneously and is given by the form

(18) $$\Omega \left(A⊗I_{CHAN}\right)\cdot \Gamma +\Omega \left(B⊗I_{CHAN}\right)\cdot \Theta +v,$$

where $I_{\mathrm{CHAN}}$ is an identity matrix of size number of fNIRS source-to-detector pairs and ⊗ is the Kronecker operator. In this way, all fNIRS source-detector pairs are analyzed simultaneously, which allows the use of the full covariance noise model (including spatial relationships) to be used in whitening the model via Equation (17). When running the higher-order statistical models, there is a program flag for including “diagnostics” as part of the toolbox. If selected, this flag will store additional model assessment information in the output variable which can be used to select outlier subjects, examine goodness-of-fit of the model, and plot the model (e.g., showing a scatter plot of how brain activity varies with age).

**Figure 4.**
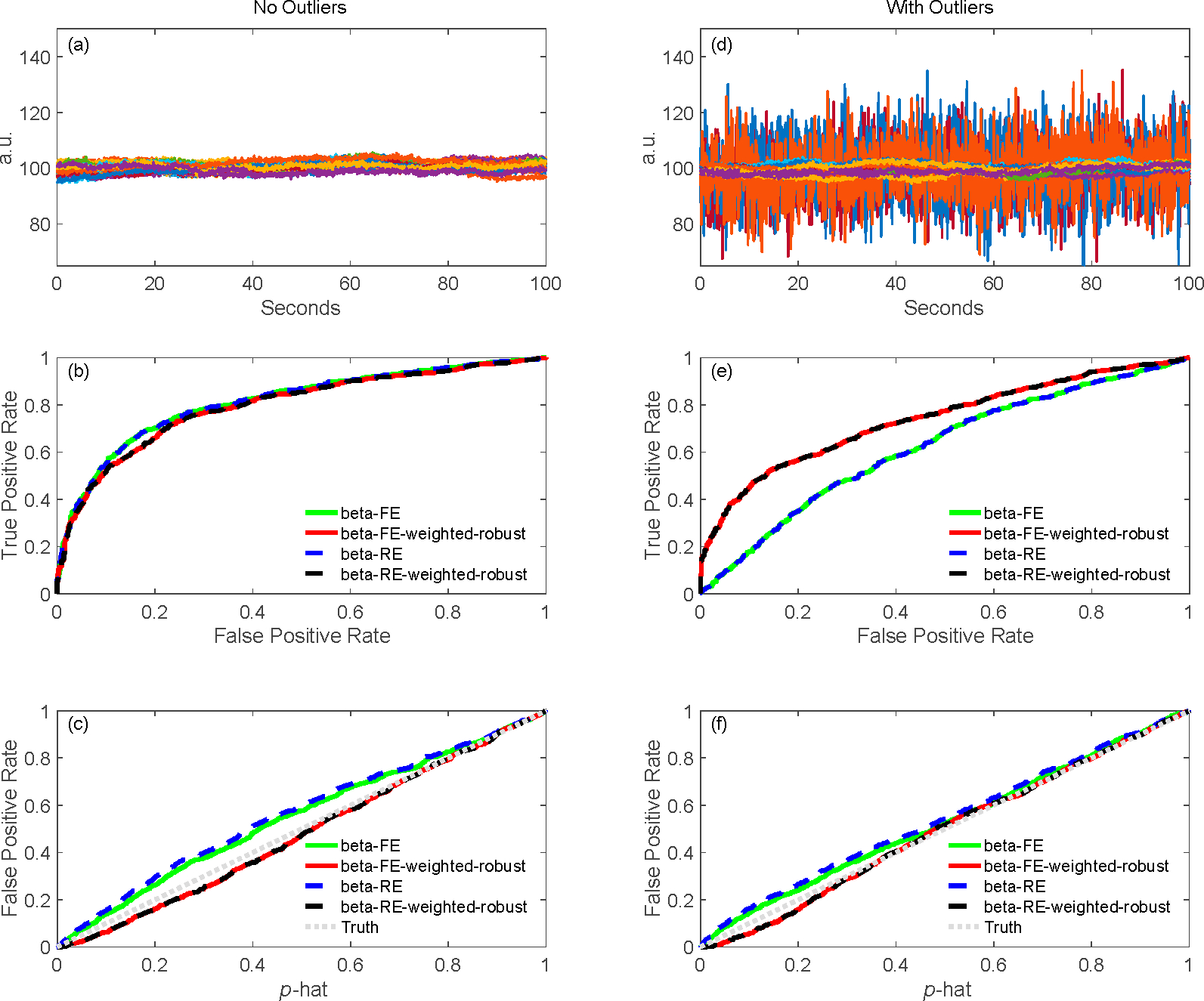
Group level comparison of no outliers (left column) and with outliers (right column). In panels (**a**,**d**), raw signals with no outliers and with outliers are shown. After simulations, the ROC curves for the various processing in the group level applied to the simulated data are shown in (**b**,**e**). Panels (**c**,**f**) show control for type-I errors for the same data and processing. An ideal curve would be along the diagonal (slope = 1), where the reported and actual FPRs would be the same.

In our second-level models, we are able to use the full covariance from the first-level model to performed weighted least-squares. This model additionally supports iterative (robust) statistics to down-weight outliers. This allows us to account for the fact that the noise in measurement channels may vary across the fNIRS probe or across subjects. In particular, contact of the optodes to the scalp will be have a large impact on the spatial variations of noise. In comparison to second level analysis using external programs such as SPSS, where either all measurements are considered to have the same noise or where the diagonal component of the noise covariance can be used to created a weighted model, by using the full covariance noise model we are able to control for the interdependence of measurements (e.g., artifacts or physiology that share noise across multiple channels). Figure 4 shows the comparison in the second-level or group-level analysis with no outliers (left column) and with outliers (right column) as shown in 4(a–c) and 4(d–f), respectively. In this simulation, we used 10 files for analyzing the second-level analysis. In the mixed effects model (see Table 3), we modeled the first-level beta values as the dependent variable with either only fixed effects (FE) or fixed and random effects (RE), combining with/without robust and covariance-weighting flags. Thirty-two channels from one file with no outliers (Figure 4a) and with 4 out of 32 channels outliers (4(b)) are shown. In this case, the outlier channels simulate a few positions with poor coupling to the scalp. In comparing Figure 4b,e, the weighted version has higher AUC in ROC curves with outliers affected for both FE and RE. For control of type-I error, all the methods have good control in the second-level analysis as shown in Figure 4c,f. As expected, when the noise across channels/subjects is uniform (Figure 4a), all models preform the same. However, when subsets of channels are simulated as noise outliers mimicking bad contact of the fNIRS sensors, then using the first-level covariance models to weight the regression produces better estimates.

## 4. Image Reconstruction Modules

Based on the optical forward model, the toolbox reconstructs the subject image by solving the underdetermined linear system between changes in concentrations of HbO_2_ and Hb in the tissue and the changes in optical density using a hierarchal Bayesian inverse model. The group-level image is reconstructed by involving random-effects. The statistical testing for the significance of the solution is also given in the image reconstruction module.

### 4.1. Optical Forward Model

Optical forward model describes the relationship between changes in concentrations of HbO_2_ and Hb in the tissue, and the changes in optical density as recorded on the surface between optical sources and detectors. The toolbox is using the implementation of optical forward model in NIRFAST toolbox [19,20] that is integrated into our toolbox as an external resource. The toolbox also interfaces with the Mesh-based Monte Carlo (MMC) [51], graphics processing unit (GPU) based Monte Carlo eXtreme (MCX) [24], and volume-based Monte Carlo (tMCimg) [26] models. A semi-infinite slab solution is also included. These forward model solvers will generate the linear optical forward model jacobian $\left(A_{i,j}\right)$ in a consistent form across all solvers. The optical forward model describes the relationship between changes in hemoglobin in the underlying brain space and the optical density measurements between optodes and is given by the expression

(19) $$\Delta OD_{i,j}^{\lambda }=A_{i,j}^{\lambda }\cdot \left[\varepsilon_{{HbO}_{2}}^{\lambda }\cdot \left(\Delta \left[{HbO}_{2}\right]+\omega_{{HbO}_{2}}\right)+\varepsilon_{Hb}^{\lambda }\cdot \left(\Delta [Hb]+\omega_{Hb}\right)\right]+v_{i,j}^{\lambda }$$

where $△OD_{i,j}^{\lambda }$ is the change in optical density, $A_{i,j}^{\lambda }$ is the jacobian of the optical measurement model corresponding to the sensitivity matrix [52], and $v_{i,j}^{\lambda }$ is an additive noise in the measurement space along the diffuse path traveled by the light between light emitter and detector pair (i,j) at the wavelength λ. $\varepsilon_{HbX}^{\lambda }$ is the molar extinction coefficient, Δ[HbX] is the change in molar concentration (fixed effect), and $\omega_{HbX}$ is an additive noise (random effect) in the image space for oxy- or deoxy-hemoglobin. Δ[HbX] and ω are vectors representing the changes at each position.

Changes in oxy- and deoxy-hemoglobin can be inferred from optical measurements at multiple (N) wavelengths by solving the forward model with spectral priors given by the expression

(20) $$\left[\begin{array}{c} \Delta OD_{i,j}^{\lambda_{1}}\\ \Delta OD_{i,j}^{\lambda_{2}}\\ \vdots \\ \Delta OD_{i,j}^{\lambda_{N}} \end{array}\right]=\left[\begin{array}{cc} A_{i,j}^{\lambda_{1}}\cdot \varepsilon_{{HbO}_{2}}^{\lambda_{1}} & {\;A}_{i,j}^{\lambda_{1}}\cdot \varepsilon_{Hb}^{\lambda_{1}}\\ {\;A}_{i,j}^{\lambda_{2}}\cdot \varepsilon_{{HbO}_{2}}^{\lambda_{2}} & {\;A}_{i,j}^{\lambda_{2}}\cdot \varepsilon_{Hb}^{\lambda_{2}}\\ \vdots & \vdots \\ A_{i,j}^{\lambda_{N}}\cdot \varepsilon_{{HbO}_{2}}^{\lambda_{N}} & {\;A}_{i,j}^{\lambda_{N}}\cdot \varepsilon_{Hb}^{\lambda_{N}} \end{array}\right]\cdot \left(\left[\begin{array}{c} \Delta \left[{HbO}_{2}\right]\\ \Delta [Hb] \end{array}\right]+\left[\begin{array}{c} \omega_{{HbO}_{2}}\\ \omega_{Hb} \end{array}\right]\right)+\left[\begin{array}{c} v_{i,j}^{\lambda_{1}}\\ v_{i,j}^{\lambda_{2}}\\ \vdots \\ v_{i,j}^{\lambda_{N}} \end{array}\right]$$

where $\lambda_{N}$ denotes the *N*-th wavelength. The optical forward model can be written in a compact form using matrix notation.

(21a) Y=H⋅(β+ω)+v

where β is used to describe the unknown values of the combination of oxy- or deoxy-hemoglobin changes in the tissue, given by

(21b) $$\beta =\left[\begin{array}{c} \Delta \left[{HbO}_{2}\right]\\ \Delta [Hb] \end{array}\right]$$

The details of the derivation from basic theories can be found in [33]. This model is then solved in the toolbox using an inverse operator.

### 4.2. Hierarchal Bayesian Inverse Models

The estimation of optical images by the inversion of Equation (21) entail an underdetermined problem where there generally are significantly less available measurements (Y) than unknown parameters (β) in the image to-be-estimated. In the toolbox, a restricted maximum likelihood (ReML) model is built and solved based on Bayesian theories. The maximization of the log-likelihood function is equivalent to maximizing the free-energy of the model and is given by the expression:

(22) $$\underset{\left\{\beta ,C_{N},C_{P}\right\}}{arg\;max}-\frac{1}{2}∥Y-H\cdot \beta {∥}_{C_{N}}^{2}-\frac{1}{2}{∥\beta -\beta_{0}∥}_{C_{P}}^{2}-\frac{1}{2}log\left|C_{N}\right|-\frac{1}{2}log\left|C_{P}\right|$$

where ${∥X∥}_{N}^{2}$ denote the weighted norm $\left({∥X∥}_{N}^{2}=X^{T}\cdot N\cdot X\right)$, $\beta_{0}$ is a prior assumed to be zero by default in the toolbox in the optical inverse model, $C_{N}$ and $C_{P}$ are the inverse of the measurement and parameter covariance matrices that are parameterized as a linear combination of symmetric prior matrices $Q_{N}$ and $Q_{p}$:

(23) $$C_{N}=\sum_{i}\Lambda_{i}\cdot Q_{N,i};C_{P}=\sum_{j}\Lambda_{j}\cdot Q_{P,j}$$

where Λ is the hyperparameter to adjust the weighting of these covariance components. The ReML model is an iterative approach that estimates both the underlying image and the hyper-parameters of the noise models (Cn and Cp). Thus, this model provides a objective estimate of the “tuning” parameters in the inverse model without additional user input (see [33] for details).

In order to solve the ReML model, the expectation-maximization (EM) algorithm is used. Firstly, in the expectation step β is estimated by solving the Gauss-Markov, which is equivalent to maximize Equation (22) with an estimated Λ is given. Then Λ is varied to maximize the quantity of Equation (22) in the maximization step. The value of optimal Λ is used in the expectation step of the next iteration. These two steps are iterated until the parameters converge. The final estimated values of β are the estimation of the oxy- or deoxy-hemoglobin changes in the tissue.

(24) $$\beta =\beta_{0}+{\left(H^{T}\cdot C_{N}^{-1}\cdot H+C_{P}^{-1}\right)}^{-1}H^{T}\cdot C_{N}^{-1}\cdot \left(Y-H\cdot \beta_{0}\right)$$

More details about derivation and covariance components can be found in Abdelnour et al. [33].

### 4.3. Group-Level Image Reconstruction

The image reconstruction code in the AnalyzIR toolbox can be used for either single subject reconstructions or as a group-level model. In the group-level model, a linear mixed effects model is used and specified using the same Wilkinson-Rogers notation as the channel space group-level statistical models. In the group-level image reconstruction, a single inverse model is simultaneously solved for all subjects and task conditions within a large-scale inverse rather than a separate inverse for each image. As detailed in our previous work [36], this allows the estimate of a single group-level image, which is based on finding a solution to the model that concurrently fits all the subjects’ data. This was previously shown to greatly improve group-level image estimates by reducing the uncertainties added by the ill-posed nature of the problem [36].

In the group-level reconstruction, the optical forward model can be represented using a three-level model and given by the following equations

Level I—Measurement level:

(25a) $$Y^{Subject}=H\cdot \beta^{Subject}+v^{Subject}$$

Level II—Subject level:

(25b) $$\beta^{Subject}=\beta^{Group}+\Delta \beta^{Subject}$$

Level III—Group level:

(25c) $$\beta^{Group}=\beta_{0}^{Group}+\omega^{Group}$$

where $\beta_{0}^{Group}$ is a prior on the expected value of the brain image for the group and the three noise terms (v,Δβ,ω) are defined as follows (${𝒩}_{N}(\mu ,\Sigma )$ denotes a N-variate normal distribution with mean vector μ and covariance matrix Σ where N is the number of measurements):

(25b) $$v^{Subject}\sim {𝒩}_{N}\left(0,C_{N}^{-1}\right)\;\Delta \beta^{Subject}\sim {𝒩}_{N}\left(0,C_{B}^{-1}\right)\;\omega^{Group}\sim {𝒩}_{N}\left(0,C_{G}^{-1}\right)$$

The free-energy expression for the model is given by

(26) $$\begin{array}{c} arg\;max\\ \left\{\beta ,C_{N},C_{B},C_{G}\right\} \end{array}\begin{array}{c} -\frac{1}{2}{∥Y-H\cdot \beta^{Subject}∥}_{C_{N}}^{2}-\frac{1}{2}{∥\Delta \beta^{Subject}∥}_{C_{B}}^{2}-\frac{1}{2}{∥\beta^{Group}-\beta_{0}^{Group}∥}_{C_{G}}^{2}\\ -\frac{1}{2}log\left|C_{N}\right|-\frac{1}{2}log\left|C_{B}\right|-\frac{1}{2}log\left|C_{G}\right| \end{array}$$

To solve the model, the covariance matrices are parameterized and ReML is implemented to solve the model, which is very similar to the inverse model in 4.2. The details can be found in [36]. In this model, all the subjects are simultaneously used to estimate the most consistent underlying image of the group-level brain activity. Our previous work [36] demonstrated that this greatly reduced the spatial point-spread introduced by probe registration and head anatomy differences and helped to avoid the effects of “blind-spots” in low density fNIRS probes by additionally using the information from all the subjects. The group-level random effects model described in Abdelnour et al. [36] is implemented in the image reconstruction code of our toolbox. In addition, this model has been extended to allow additional covariates and terms similar to the channel-level mixed effects model discussed in Section 3.5. Our image reconstruction model uses the same Wilkinson–Rogers’ notation as the channel-level version.

### 4.4. Statistical Testing

The toolbox also provides statistical testing for the significance of image space estimates of brain activity. For, the *j*-th voxel, the null and alternative hypotheses are:

(27) $$H_{0}:\beta_{j}=0;H_{1}:\beta_{j}\neq 0$$

where $\beta_{j}$ is the *j*-th element of β. Previous study [53] describes a method to perform significance testing in ridge regression whose solution is a special condition of Equation (24). A similar Student *t*-test is used in the toolbox for the significance testing. The test statistic is defined as

(28) T=β^jseβ^j

where β^j is the estimate of $\beta_{j}$ and seβ^j is the estimate of its standard error. Analogous to the previous study [53] se β^j is obtained as the square root of the *j*-th element of the diagonal of the covariance matrix

(29) Covβ^=σ2HT⋅CN-1⋅H+CP-1-1⋅HT⋅CN-1⋅CN-1T⋅H⋅HT⋅CN-1⋅H+CP-1-1

In practice, $\sigma^{2}$ is estimated using the residual mean square of the model given by

(30) σ^2=(Y-H⋅β^)′(Y-H⋅β^)v

Here v is the residual effective degrees of freedom that can be calculated by $v=N-tr\left(2H-H\cdot H^{\prime }\right)$ (tr() returns the trace of the matrix) and H is the “hat matrix” defined as

(31) $$H=H\cdot {\left(H^{T}\cdot C_{N}^{-1}\cdot H+C_{P}^{-1}\right)}^{-1}\cdot H^{T}\cdot C_{N}^{-1}$$

Under $H_{0}$, T follows a Student t distribution with N-tr(H) degrees of freedom. Then the statistic and degrees of freedom can be used to report the p-value for the significance of $\beta_{j}$. In order to save memory in the code, the Cholesky decomposition of the covariance matrix Equation (17b) is stored instead of the full model and the full elements are computed as needed.

## 5. Connectivity and Hyper-Scanning Modules

Two main challenges for spontaneous functional connectivity fNIRS (sFC-fNIRS) analysis are the slow temporal structure of both systemic physiology and blood vessels, and motion related artifacts from movement of the fNIRS sensors on the participants’ head. In order to protect the sFC-fNIRS from false-discoveries, the noise model needs to be generalized to account for auto-correlative errors and motion-related outliers from a normal distribution. The approach is similar with the general linear model for time series regression analysis of evoked signals chances (e.g., [15,20]). However, the GLM approach in fNIRS model requires additional steps or higher order corrections than those often used in other modalities (e.g., fMRI), because the properties of noise in fNIRS are different and the fNIRS is typically temporally over-sampled compared to the slower physiological signals. We have previously detailed and compared these approaches for evoked time-series analysis of fNIRS [15]. In this section, we will briefly describe the similar pre-conditioning of fNIRS for sFC connectivity to deal with statistical outliers in a linear model. The models used in the AnalyzIR toolbox are further detailed in our previous work [18].

### 5.1. Correlation Models

The traditional approach to connectivity analysis is to simply compute the Pearson correlation between all possible pairs of channels. However, artifacts arising from systemic physiological noise and head motion necessitate special considerations [18].

#### 5.1.1. Pre-Whitening

Physiological noise in the fNIRS signals are temporally correlated (colored) noise. In this case, pre-whitening filter removes the autocorrelation and whiten the frequency content of the signal. A pre-whitening filter can be defined from an autoregressive model of the data. The effect of this approach on fNIRS signals has been presented in our previous work [16]. Once applied to the data, yields an uncorrelated innovations signal. The autoregressive model for a signal (Y) is defined as

(32a) $$Y_{\left\{t\right\}}=\sum_{i=1}^{P}a_{i}\cdot Y_{\left\{t-i\right\}}+\epsilon_{\left\{t\right\}}$$

(32b) $$\epsilon_{\left\{t\right\}}\in N\left(0,\sigma^{2}\right)$$

where t indicates the sample point (time) and the set $a_{i}$ is the autoregressive coefficients of the model, which need to be estimated. P is the model order, which can be selected using an information criteria such as BIC (Bayesian information criteria) [54]. Equations (32a) and (32b) says that the current sample point $\left(Y_{\left\{t\right\}}\right)$ can be predicted based on the last several time-points in its history $\left(a_{1}\cdot Y_{\left\{t-1\right\}}\ldots a_{p}\cdot Y_{\left\{t-1\right\}}\right)$ and newly added information at that time point, which is called the innovations $\left(\varepsilon_{\left\{t\right\}}\right)$. The innovations can be thought of as the new information that is added to the total signal at each time point. The innovations time-course is a whitened signal with no autocorrelation representing the signal information added at each time point. The innovations signal can be estimated by first fitting the autoregressive coefficients of the model and using them to filter the original signal. Pre-whitening is applied to any two signals A and B to yield their respective whitened innovations models $A_{w}$ and $B_{w}$. Instead of correlating the original signals, the two innovations ($A_{w}$ and $B_{w}$) are compared which estimates the correlation of the addition of only the new information being added to both signals at each time point. So-called Wiener causality models [55] are an implementation of this concept that specially look at the relationship of lagged cross terms in the innovations (e.g., the history of signal B predicts the current value of A). In the remainder of this section however, we focus on the zeroth lag correlation terms.

The pre-whitening will remove the serially correlated errors between two signals for both no motion and motion affected confirming. After pre-whitening using an appropriate high model order, the autocorrelation of both motion-affected and –unaffected drops to random change within a single time point. The comparison of the analysis of original data, whitened signal, and autocorrelation have been examined in depth in Santosa et al. [18] (see Figure 2 in [18] for detail).

#### 5.1.2. Robust Methods

Pre-whitening via autoregressive removes serial correlations and autocorrelations between sample points. However, the whited signals will still contain outliers (e.g., heavy-tailed noise) due to motion artifacts [15]. Robust regression methods or pre-weighting is an approach for dealing with statistical outliers in a linear model through iteratively estimating the residual noise of the model using a weighted least-squares fit and computing the weight based on outliers in the residual. Previously, this model as applied to reduce the effect of motion in fNIRS functional data [16]. Robust regression is a regression method in which one signal is considered the data and the other is considered the regression. This approach would fix leverage from motion-artifacts related that act independently between channels. We have detailed the use of robust regression for investigating evoked signals in fNIRS in several our previous publications [15,16]. We propose a joint weighting matrix, which is computed from the geometric length of both time-courses in order to reduce outliers and normalize the noise distributions. Thus, we propose to compute the correlation between two signals, which are now the two weighted and pre-whitened signals. The weighting function used in this work is given by Tukey’s bisquare function [45] and is the same model as used in Barker et al. [16] (see Equation (4) in [16]).

### 5.2. Coherence Models

In addition to correlation models, wavelet coherence can be used to define connectivity [56]. The continuous wavelet transform (CWT) of a signal is a representation of the power contained at each time and frequency point. This is achieved using a time- and frequency-localized function known as a wavelet. One common choice is the Morlet wavelet, defined as

(33) $$\psi_{0}(\eta )=\pi^{-\frac{1}{4}}e^{i\omega_{0}\eta }e^{-\frac{1}{2}\eta^{2}}$$

where $\omega_{0}$ is dimensionless frequency and η is dimensionless time. The continuous wavelet transform is then computed by convolving the input signal with wavelets at different time-scales, resulting in a time by frequency matrix of signal power. The CWT (W) for a signal x at times n=1…N is defined as

(34) $$W_{t}\left(s\right)=\sqrt{\frac{\delta t}{s}}\sum_{t^{\prime }=1}^{T}x_{t^{\prime }}\psi_{0}\left[\left(t^{\prime }-t\right)\frac{\delta t}{s}\right]$$

where δt is the sampling period, and *s* is the time scale. This principle can be extended to connectivity by computing the cross-wavelet transform of two input signals, e.g., the product of the CWT of one signal and the complex conjugate of another $\left(W_{XY}=W_{X}W_{Y}^{*}\right)$. The power of cross-wavelet transform is then normalized by the power of the CWTs for each signal to yield the wavelet transform coherence $\left(R^{2}\right)$:

(35) $$R^{2}=\frac{{\left|W_{XY}\right|}^{2}}{{\left|W_{X}\right|}^{2}{\left|W_{Y}\right|}^{2}}$$

The AnalyzIR implementation of wavelet coherence differs from other toolboxes in using robust methods for estimating the wavelet transform and high-order AR pre-whitening to control type-I error due to serial correlations in the data [18]. Currently only the Morlet wavelet is implemented based on previous fNIRS studies [56], however other options may be explored in future work.

### 5.3. Hyperscanning

The toolbox contains a module for hyperscanning analysis. Hyperscanning is a form of fNIRS where two or more subjects are simultaneously recorded during an interactive task and the brain signals between the subjects is analyzed. For example, if two subjects are talking to each other, the Wernike’s and Broca’s brain regions would be expected to correlate. In the toolbox, the hyperscanning module requires a user-defined table of subject pairs. This table is automatically populated in the case of NIRx data files collected with a hyperscanning configuration. The module also requires specification of the analysis model, which can be any of the correlation, wavelet coherence, or Granger’s causality models previously described. Finally, the module has a flag for forcing symmetry in the estimate. Symmetry is imposed to prevent arbitrary assignment of person “A” and “B” in the hyperscanning model. This is modeled by combining the results for A→B and B→A. For example, in the case of a parent-child interaction, the parent might have more regions in their brain “connected” to the child’s than the child does to the parent. To test this, the symmetry flag should be set to false in the model since the definition of the parent (“A”) and the child (“B”) is not arbitrary. However, in the case of two adults, these definitions could be arbitrary and the option is given in the code to allow either approach.

### 5.4. Group Connectivity Models

A processing module for fixed and mixed effects group-level connectivity analysis is offered in the toolbox. This model is similar to the implementation used in activation analyses and similarly supports Wilkinson-Roger’s notion allowing for complex linear and interaction models to be defined. For first-level correlation model inputs, the correlation value is Fisher Z-transformed as the input to the analysis. The model is then carried out for each unique connection in the connectivity matrix. Calculation of Benjamini-Hochberg FDR-corrected *p*-values is done in a manner to take advantage of the symmetry inherent in undirected connectivity matrices, as well as that introduced by imposing symmetry to undirected hyperscanning models.

### 5.5. Graph-Models

An additional method of connectivity analyses is known as complex network analysis on graph theory. This approach entails defining each measurement channel as a node in a graph, and the relationship between any two measurements as an edge. The edges may be derived from correlation, coherence, or any other similarity metric, and are often thresholded to produce a binary graph of connected and disconnected nodes. Once in this form, a number of algorithms can be employed to determine network-level characteristics, such as node clustering, network efficiency, and small-worldness (the ratio of clustering to mean distance between nodes). The graph theoretic approach allows large connectivity matrices to be characterized by a small number of scalar parameters, making it particularly useful for the development of neural biomarkers. The AnalyzIR toolbox supports the conversion of both first and higher-level connectivity (and hyperscanning) statistical models to graph-based models which are compatible with the Brain Connectivity Toolbox [57].

**Figure 5.**
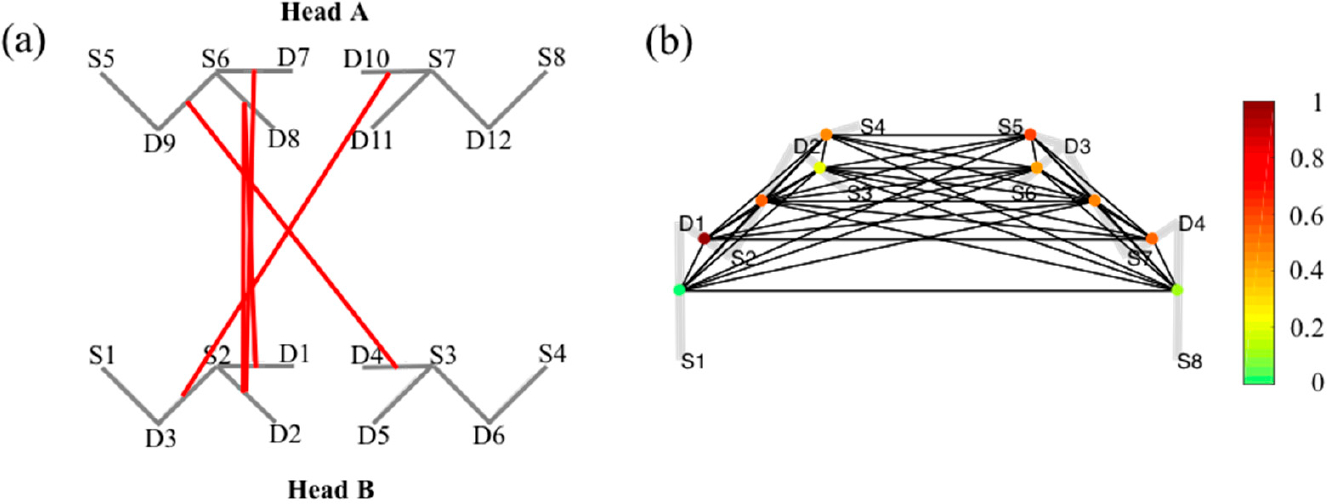
Examples of connectivity analyses employed by AnalyzIR: (**a**) Connectivity between two subjects during cooperation on a puzzle task; (**b**) relative PageRank centrality for a single subject.

Figure 5a demonstrate the connectivity of simultaneously measured brain activity using fNIRS on two subjects (A and B) during a cooperative puzzle-solving task. The toolbox provides facilities to calculate and visualize connectivity between two or more subjects. The page rank centrality of a single subject is shown in Figure 5b. Page rank centrality is a variant of eigenvector, which was developed by Google search to rank websites in their search engine results [58–60]. In brain network, researchers use this technique to describe the connectivity patterns of nodes (or channels) and links of a network based on adjacency matrix. The color bar in the right corner shows the degree of connection.

## 6. Toolbox Utilities

In addition to processing modules, the toolbox offers additional utilities for probe registration and adjusting for head size, region-of-interest analysis, and other features.

### 6.1. Probe Registration

The *nirs.core.Probe* object class encodes both two-dimensional and three-dimensional information about the fNIRS sensor layout. Three-dimensionally registered information can be imported directly from the raw data files (e.g., the case in NIRx data file format) or using third-party code such as AtlasViewer [61]. Within the toolbox there are features to register the probe. To register a fNIRS probe a set of landmarks need to be defined relative to the fNIRS sensors in the two-dimensional space. For example, the 10–20 point FpZ might be located in between two specific optodes on the probe. These landmarks are defined as either “anchors” or “attractors” and can be any of the standard 10–20, 10–10, or 10–5 head point coordinates. Anchors define landmarks that are fixed in position relative to the fNIRS optodes. Attractors define directional information. For example, the *y*-axis of the fNIRS probe might be oriented in the direction of the 10–20 points FpZ to Cz. This notation is similar to the use in the AtlasViewer program [61]. The probe is then registered to a generic spherical shape using an interactive relaxation of a network of connected springs between the optodes. In our toolbox, the springs connecting optodes are automatically populated (c.f. AtlasViewer where this is user-defined). For disjointed probes (e.g., where the probe on the left/right hemispheres are separate pieces), an option is included to separate register each part as long as each piece has at least one anchor point. In registering the probe to the spherical shape, the size of the shape (head) can be changed by specifying the distance over the top of the head from the nasion to inion, left/right perarticular points, and the head circumference. If only one of the three distances is specified, then the shape is isotropically stretched.

Once the probe is registered into a 10–20 (spherical) space, a head/brain model can be registered into the same space. A version of the Colin27 atlas [62] is included in the toolbox, which has been preprocessed using FreeSurfer [63] to generate a layered head model (skin, skull, cerebral spinal fluid, gray/white matter). The gray/white matter surfaces have been labeled using both the AAL2 [64,65] and Freesurfer “aparc” [66] parcellation labels which is stored in the variable. Subject specific models can also be imported from FreeSurfer or similar programs. The registration of the fNIRS probe to the brain model can either be done by rescaling the brain to match the measured head circumference of the subject or by re-registering the probe to match the measurements computed from the brain/head model.

Once registered, the probe object’s drawing is controlled through the *default_draw* field, which provides the choose of the original two-dimensional (“2D”), in 10–20 space (“10–20”; which uses a Clarke azimuthal map projection), or as a three-dimensional drawing overlain on the head mesh “3D mesh(<view>)”. The keyword “zoom” added to the end of the draw field string will cause the view to be zoomed to show only the part of the map covered by the probe (c.f. showing the whole 10–20 map as demonstrated back in Figure 1).

### 6.2. Depth-Maps

**Figure 6.**
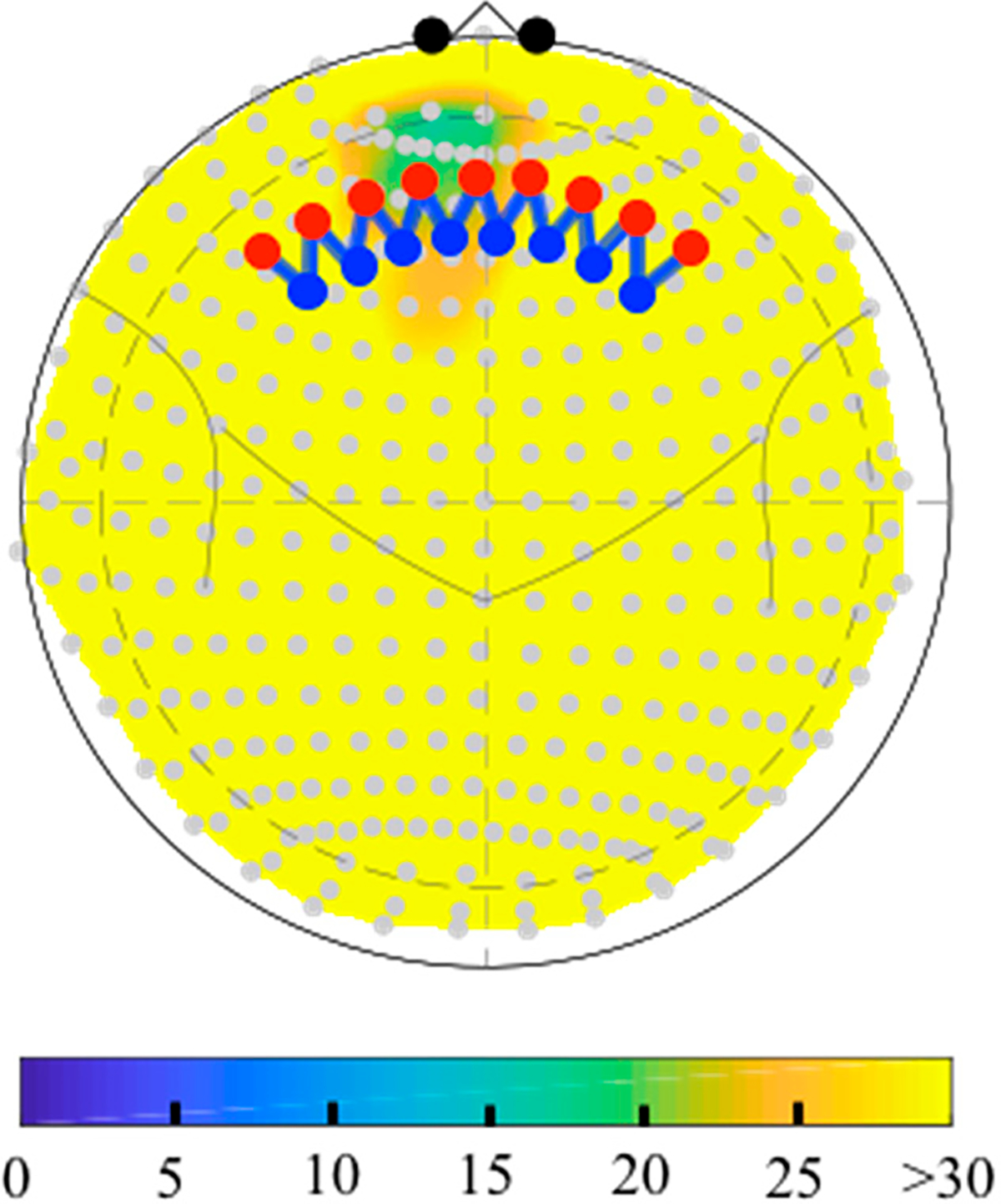
The fNIRS probe was registered to the Colin27 atlas, which was used in combination with the automatic anatomical labeling toolbox (AAL2) to label the Brodmann area 10. The images above show topology maps (Clarke azimuthal map projection) showing the depth of the nearest cortical point in the region-of-interest to the surface of the head. A yellow indicates a depth of greater than 30 mm, which would be inaccessible to fNIRS.

The depth map function (*nirs.util.depthmap*) will compute the distance from each fNIRS probe position (sources, detectors, and midpoint of the measurements) to the labeled anatomical pial surface of the brain based on the registered head model. This can either be returned in table form or plotted as a figure as shown in Figure 6. This function can be used to estimate the depth to a specific brain region using labels from the parcellations stored brain model (e.g., “BA-46”; for the toolbox included Colin27 atlas these can be any of the AAL2 or Freesurfer labels) or to return the closest region to each fNIRS sensor using the “?” keyword. The depth map table also includes an estimate of the relative sensitivity of each measurement to the brain region, which is based on a quick homogeneous slab-model version of the forward solution (see Section 4).

### 6.3. Region of Interest Analysis

The toolbox includes support for region-of-interest analysis. Region-of-interest analysis can be applied to either the time course (*nirs.core.Data*) or statistical model (*nirs.core.ChannelStats*) variables. The region-of-interest code returns an object of the same form as either the time-course or *ChannelStats* variables and can be used in the processing modules. Similar to computing the Student’s *t*-value over several task conditions, region-of-interest analysis is done via the equation

(36a) $$\beta_{roi}=c\cdot \beta_{channel}$$

(36b) $${Cov}_{\beta_{roi}}=c\cdot {Cov}_{\beta }\cdot c^{T}$$

where c is the contrast vector and β is the channel-space values (either from the ChannelStats model or as channel-by-time matrices for the time-series data). In a simple version, the contrast vector of $c={\left[00.50.500\ldots \right]}^{T}$ would average over the 2nd and 3rd channel entries.

Region-of-interests can be defined using tables defined by the list of the sources, detectors, and weights used. This can be specified manually or based on anatomical registration. For three-dimensionally registered probes, the expected relative sensitivity of each fNIRS source-to-detector channel to anatomical parcellation labels can be used to define a weighted region-of-interest based on the optical forward model (H). The optical forward model (see Section 4) defines the sensitivity of the measurements in channel space to underlying changes in the brain space. This model is calculated by estimation or simulation of the diffusion of light through the tissue (see [67] for details).

For anatomical regions-of-interest, one can use the optical forward model and a brain-space region mask define the contrast vector in channel-space. The contrast vector (c) is given by

(37a) $$c_{roi}=H\cdot Mask_{ROI}$$

where

(37b) $${Mask}_{ROI}(r)=\left\{\begin{array}{cc} 1 & \;\text{if}\;r\in ROI\\ 0 & else \end{array}\right.$$

The Studenťs t-test for a specific region-of-interest is then given by

(37c) $$T_{ROI}=c\cdot \Gamma /\sqrt{c\times {Cov}_{\Gamma }\times c^{T}}$$

and

(37d) $$c=c_{COND}\cdot c_{ROI}$$

where $c_{ROI}$ and $c_{COND}$ are the contrast vectors for the region-of-interest (ROI) and for the pooling of conditions. For statistical testing of the region-of-interest, this contrast vector defines the expected response in channel space given the region in brain space. Thus, Equation (37) test the null hypothesis that the signal from the region-of-interest is equal to zero. This does not test if the activity specifically came from only that region. In other words, this does not test if the entire region is active or just a subset. It also does not rule out that the activity could have been from a nearby region, which was also covered by that source-detector pair. This also assumes the optical forward model and probe registration are accurate. Any mismatch in the registration or forward model (e.g., as a result of anatomical differences including brain atrophy) will mean that the contrast vector is testing a slightly non-optimal hypothesis. As discussed in [15], using a sub-optimal contrast vector for the hypothesis is equivalent to using the wrong time-window for computing the effect. In this case, it is akin to a weighted average across the wrong combination of channels. This introduces type-II error (e.g., the false-negative rate will increase and one might miss activity that actually was significant), but does not introduce type I error (false-positive rate).

### 6.4. Regression Testing

One of the key features of the toolbox is the ability to quantitatively compare analysis methods and pipelines. Datasets with and without stimulus are simulated and analyzed using different pipelines. Receiver operating characteristics (ROC) curve is drawn for each pipeline given the analysis results of the simulation data, which will be used in the comparison of different pipelines.

#### 6.4.1. Data Simulation

The channel space noise and image space stimulus are generated in the data simulation. The noise appears in all channels and the stimulus only generated within a specific ROI. Two datasets are generated in every simulation iteration with one of which contains a simulated evoked response and the other one only contains noise (null data).

##### Noise Generation

An autoregressive (AR) model is used to generate the noise. The notation AR(p) indicates an AR model of order p, which is defined as

(38a) $$Y_{t}=c+\sum_{i=1}^{p}\varphi_{i}Y_{i-1}+\varepsilon_{t}$$

where $\varphi_{1},\cdots ,\varphi_{p}$ are the randomly generated parameters of the model, c is a constant representing the baseline offset which is 0 in the toolbox for simulating changes in optical density, and $\varepsilon_{t}$ is the noise. In our case, noises of different channels are correlated. Thus, ε is sampled from a N-variate normal distribution as follows.

(38b) $$\varepsilon_{t}\sim {𝒩}_{N}(0,S)$$

Here $\varepsilon_{t}$ is the N×T dimensional noise matrix, in which N is the number of channels, T is the total simulation time, and S is the N×N dimensional covariance matrix between channels.

##### Stimulus Generation

The time difference between two neighboring stimulus onsets is generated from an exponential distribution with a specific mean that indicates the average difference. The responses (changes in HbO_2_ and Hb) of the brain at stimulus onsets are simulated using the function given by Equation (15).

#### 6.4.2. ROC Definitions

There are two levels ROC [68] curves that are produced by the toolbox – voxel level and ROI level. The *p*-values for each voxel within the ROI and for the whole ROI in stimulus present data and noise only data are used as the decision variable. The logic is that a smaller *p*-value indicates more significant changes in HbO_2_ and Hb, i.e., stimulus present. Then, sort the *p*-values and use each unique value as the threshold. The false positive rate (FPR) and true positive rate (TPR) at each threshold can be calculated by the fraction of the voxels/ROIs with a *p*-value that is smaller than or equal to the threshold using stimulus present and noise-only dataset respectively. The ROC curve is defined as TPR against FPR. The area under the ROC curve (AUC) [68] means the probability that the HbO_2_ and Hb changes in stimulus-containing voxels/ROIs are more significant than that in noise-only voxels/ROIs based on the estimations given by the pipeline. Therefore, a larger AUC indicates higher detection accuracy.

The statistical definition of *p*-value is the probability of rejecting the null hypothesis when it is true. Hence, we use *p*-value as an estimate of the FPR. Another two plots the toolbox draws are defined by FPR against *p*-values for voxels and ROIs respectively. A good curve should be close to the diagonal of the plotting square. These two curves indicate the bias of the FPR estimates by the pipeline. The curve above the diagonal means the FPR is underestimated and vice versa.

## 7. Graphical Interfaces

**Figure 7.**
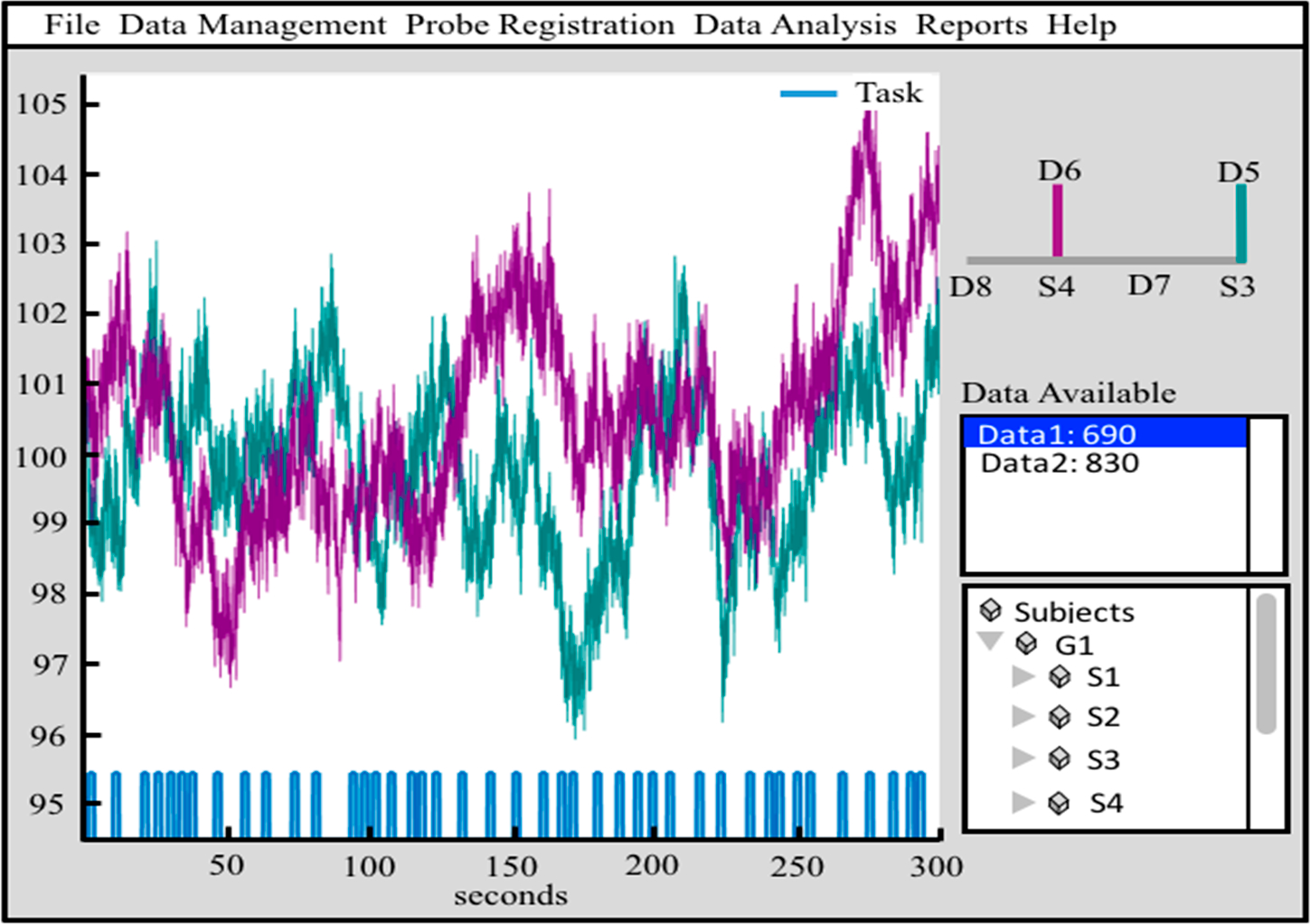
Graphical user interface of AnalyzIR toolbox.

Although the Brain AnalyzIR toolbox is primarily a command-line interface, there are available several graphical user interfaces (GUIs) (e.g., *nirs.viz.jobmanager;* a tool for building analysis pipelines, *nirs.viz.nirsviewer*; a data display utility, and *nirs.viz.StimUtil*; a tool for modifying stimulus information). Figure 7 shows the screenshot of *nirs.viz.nirsviewer* to visualize the time series of raw data (690 nm) from two channels with its stimulus info. The menu commands (i.e., File, Data Management, Probe Registration, Data Analysis, Reports, Help) provide access to most operations available in the toolbox through the graphical interface for users who prefer not to use the command line. For example, this GUI will provide the ability to load NIRS files, edit subject demographics, register probe, etc. The GUI also provides access to data structures (e.g., raw, wavelength, hemoglobin data, etc.) and NIRS files (subject information from demographics). The stimulus design and signal from a particular channel can be viewed by selecting the corresponding source-detector pairs in the probe configuration. In Figure 7, it shows two channels (channels from source 4—detector 6 and source 3—detector 5) from 300 s data of 690 nm with the stimulus design of the task (“Task”).

## 8. Minimum Processing Recommendations

Our recommendation for standard analysis of fNIRS data is to do the minimum amount of manipulations to the data possible. In particular, by using statistical models that are more robust to the effects of physiology (serially-correlated errors and colored-noise) and motion-artifacts (statistical outliers), we can control false-positive rates even without removing or pre-processing these artifacts ahead of time. Thus, our preferred analysis pipeline does not include any motion-correction or pre-filtering of any kind and instead focuses on using appropriate statistical models that are less biased by these artifacts. Of course, the presence of these artifacts and noise does affect the effect sizes and statistical power of the results, resulting in increased type-II error. Using pre-filtering or correction methods will generally improve the size of the estimated effects, however, there is rarely a “one-size-fits-all” solution to these pre-processing issues. In contrast, using these robust statistical models does work for dealing with the issues seen in fNIRS data that often result in high false-positive rates.

In the toolbox, there are a series of default module pipelines that represent standard minimum processing. These are found in *nirs.modules.default_modules* and include a single-subject canonical model analysis, a single-subject deconvolution (FIR) model analysis, and a pipeline from raw data through group-level mixed effects models. These pipelines only include standard steps like the conversion to optical density, the modified Beer–Lambert law, resampling to the default (5Hz), and linear regression modeling using the AR-IRLS method.

## 9. Future Direction

FNIRS technology will continue to evolve alongside other modalities to improve our understanding of human brain function. This open-source toolbox allows other researchers to freely use, modify, or share, while respecting the original toolbox authorship. We welcome more interaction with other researchers from various modalities (e.g., fNIRS, fMRI, MEG, EEG) on this AnalyzIR toolbox with a particular focus on developing multimodal methods within this common framework. The toolbox already supports all four of these modalities in some form. It is our hope that this toolbox will continue to grow and advance with (but not restricted to) the NIRS field. In addition, we already implemented the modules of the short-separation measurements in the toolbox as a popular technique to reduce the systemic physiological noises in the fNIRS signal. We also encourage researchers to use ROC analysis when they are proposing new methods. Doing so will allow proper comparisons of the performance of the proposed method with existing method.

## Figures and Tables

**Table 1.** List of the core data classes in the AnalyzIR toolbox.

Class	Purpose	Methods	Description
*nirs.core.Data*	Holds time-series information including stimulus events	*< >.draw([channel index])*	Draws the time-course of a channel of data
*nirs.core.Probe*	Holds information about	*< >.draw()*	Draws the layout of the probe in 2D or 3D
	the probe design and	*<> .default_draw_function*	Sets the default draw behavior on 3D registered probes
	registration	*< >.link*	A table describing the connections of source-detector pairs
		*< >.optodes*	A table describing the source-detector and any additional probe points
*nirs.core.ChannelStats*	Holds the statistical maps in	*< >.draw(type,range,alpha)*	Draws the statistical map according to the probe
	first and second-level	*< >.table*	Returns a formatted table of the statistical values
	analysis	*< >.ttest(conditions)*	Performs a student’s *t*-test to compare two or more contrasts
		*< >.jointTest()*	Returns a FChannelStats variable for the T^2 test using HbO_2_/Hb
		< >.printAll(*, outfolder, imagetype)	Draws and saves the figures in TIFF or JPEG format
		*< >.sorted*	Returns sorted stats by columns in variables
*nirs.core.ChannelFStats*	Holds F-statistics in channel	*< >.draw(range, alpha)*	Draws the statistical map according to the probe
	Space	*< >.table*	Returns a table of all channel wise stats
		*< >.getCritF*	Returns critical F value
*nirs.core.ImageStats*	Holds the statistics for reconstructed images	*< >.draw(type, range, alpha, beta, [power])*	Draws the statistical map according to the probe
		*< >.jointTest()*	Performs a joint hypothesis test across all channels in each source-detector pair
*nirs.core.sFCStats*	Holds connectivity and	*< >.draw*	Draws the correlation values
	hyper-scanning statistical	*< >.table*	Returns a table of all stats
	models	*< >.graph*	Returns a graph object from the connectivity model

**Table 2.** List of the basic processing modules in the AnalyzIR toolbox.

Modules	Description	Citation
**Pre-processing**		
BeerLambertLaw	Converts optical density to hemoglobin	[34]
Resample	Nyquist filter and resample the data	Matlab: *resample.m* function
OpticalDensity	Conversion of raw data to optical density	
**Data management**		
AddDemographics	Add subject information from the table	
ChangeStimulusInfo	Change stimulus info to data given a table	
DiscardStims	Removes specified stimulus conditions from design	
FixStims	Modify onset/duration/amplitude of stimulus	
KeepStims	Removes all stimuli except those specified	
RemoveStimLess	Discard data files with no stimulus information	
**Filter**		
BaselineCorrection	Motion-correction filter to remove DC sifts	See Section 2.2.2.1
PCAFilter	PCA filter for motion or physiology	[19]
WaveletFilter	Filter to remove outliers and low-frequency characteristics	[35]
**Statistical analysis**		
ANOVA	Group-level ANOVA model	Matlab: *fitlme.m* function
AR-IRLS	GLM analysis using autoregressive model	[16]
Connectivity	Computes all-to-all connectivity model	[18]
Hyperscanning	Computes all-to-all connectivity between two files	[18]
ImageReconstruction	Subject or group-level image reconstruction model	[33,36,37]
MixedEffects	Group-level linear mixed effects model	Matlab: *fitlme.m* function
NIRS-SPM	GLM analysis using NIRS-SPM	[20]
OLS	GLM analysis using ordinary least squares	[19]
RemoveOutlierSubjects	Flags and removes outlier subjects based on leverage	
SubjLevelStats	Subject-level analysis	Matlab: *fitlme.m* function
**Additional**		
HOMER2	Interface to HOMER2 code	[10,19]

**Table 3.** Examples of Wilkinson–Rogers notation.

Formula	Interpretation
beta ~−1 + cond + (1|subject)	Effect of condition, controlling; for subject
beta ~−1 + group:cond + (1|age)	Effect of condition for each group, controlling for age
beta ~−1 + group + cond + group*cond + (1|IQ)	Main effects of group) and condition, and r group × condition interaction, controlling for IQ
